# SURVIV for survival analysis of mRNA isoform variation

**DOI:** 10.1038/ncomms11548

**Published:** 2016-06-09

**Authors:** Shihao Shen, Yuanyuan Wang, Chengyang Wang, Ying Nian Wu, Yi Xing

**Affiliations:** 1 Department of Microbiology, Immunology and Molecular Genetics, University of California, Los Angeles, Los Angeles, California 90095, USA; 2 Department of Molecular and Medical Pharmacology, University of California, Los Angeles, Los Angeles, California 90095, USA; 3 Bioinformatics Interdepartmental Graduate Program, University of California, Los Angeles, Los Angeles, California 90095, USA; 4 Department of Statistics, University of California, Los Angeles, Los Angeles, California 90095, USA

## Abstract

The rapid accumulation of clinical RNA-seq data sets has provided the opportunity to associate mRNA isoform variations to clinical outcomes. Here we report a statistical method SURVIV (Survival analysis of mRNA Isoform Variation), designed for identifying mRNA isoform variation associated with patient survival time. A unique feature and major strength of SURVIV is that it models the measurement uncertainty of mRNA isoform ratio in RNA-seq data. Simulation studies suggest that SURVIV outperforms the conventional Cox regression survival analysis, especially for data sets with modest sequencing depth. We applied SURVIV to TCGA RNA-seq data of invasive ductal carcinoma as well as five additional cancer types. Alternative splicing-based survival predictors consistently outperform gene expression-based survival predictors, and the integration of clinical, gene expression and alternative splicing profiles leads to the best survival prediction. We anticipate that SURVIV will have broad utilities for analysing diverse types of mRNA isoform variation in large-scale clinical RNA-seq projects.

## Abstract (web summary)

Clinical RNA-seq datasets can predict clinical outcomes. Here, Shen *et al*. report a statistical method for survival analysis of mRNA isoform variation using clinical RNA-seq datasets, and the identified isoform based survival predictors outperform gene expression based survival predictors using TCGA data on six cancer types.

## Graphical abstract

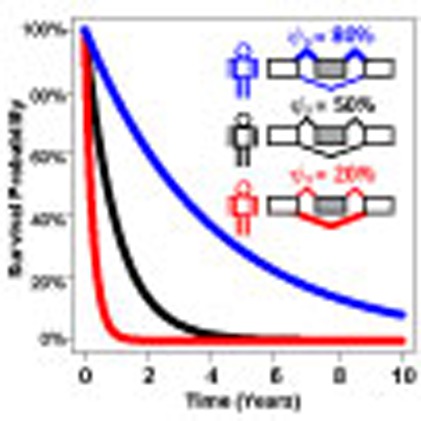

Eukaryotic cells generate remarkable regulatory and functional complexity from a finite set of genes. Production of mRNA isoforms through alternative processing and modification of RNA is essential for generating this complexity. A prevalent mechanism for producing mRNA isoforms is the alternative splicing of precursor mRNA^1^. Over 95% of the multi-exon human genes undergo alternative splicing^2^,^3^, resulting in an enormous level of plasticity in the regulation of gene function and protein diversity. In the last decade, extensive genomic and functional studies have firmly established the critical role of alternative splicing in cancer^4^,^5^,^6^. Alternative splicing is involved in a full spectrum of oncogenic processes including cell proliferation, apoptosis, hypoxia, angiogenesis, immune escape and metastasis^7^,^8^. These cancer-associated alternative splicing patterns are not merely the consequences of disrupted gene regulation in cancer but in numerous instances actively contribute to cancer development and progression. For example, alternative splicing of genes encoding the Bcl-2 family of apoptosis regulators generates both anti-apoptotic and pro-apoptotic protein isoforms^9^. Alternative splicing of the *pyruvate kinase M* (*PKM*) gene has a significant impact on cancer cell metabolism and tumour growth^10^. A transcriptome-wide switch of the alternative splicing programme during the epithelial–mesenchymal transition plays an important role in cancer cell invasion and metastasis^11^,^12^.

RNA sequencing (RNA-seq) has become a popular and cost-effective technology to study transcriptome regulation and mRNA isoform variation^13^,^14^. As the cost of RNA-seq continues to decline, it has been widely adopted in large-scale clinical transcriptome projects, especially for profiling transcriptome changes in cancer. For example, as of April 2015 The Cancer Genome Atlas (TCGA) consortium had generated RNA-seq data on over 11,000 cancer patient specimens from 34 different cancer types. Within the TCGA data, breast invasive carcinoma (BRCA) has the largest sample size of RNA-seq data covering over 1,000 patients, and clinical information such as survival times, tumour stages and histological subtypes is available for the majority of the BRCA patients^15^. Moreover, the median follow-up time of BRCA patients is ∼400 days, and 25% of the patients have more than 1,200 days of follow-up. Collectively, the large sample size and long follow-up time of the TCGA BRCA data set allow us to correlate genomic and transcriptomic profiles to clinical outcomes and patient survival times.

To date, systematic analyses have been performed to reveal the association between copy number variation, DNA methylation, gene expression and microRNA expression profiles with cancer patient survival^16^,^17^. By contrast, despite the importance of mRNA isoform variation and alternative splicing, there have been limited efforts in transcriptome-wide survival analysis of alternative splicing in cancer patients. Most RNA-seq studies of alternative splicing in cancer transcriptomes focus on identifying ‘cancer-specific' alternative splicing events by comparing cancer tissues with normal controls (see refs 18, 19, 20, 21, 22, 23 for examples). A recent analysis of TCGA RNA-seq data identified 163 recurrent differential alternative splicing events between cancer and normal tissues of three cancer types, among which five were found to have suggestive survival signals for breast cancer at a nominal *P*-value cutoff of 0.05 (ref. 21). Some other studies reported a significant survival difference between cancer patient subgroups after stratifying patients with overall mRNA isoform expression profiles^24^,^25^. However, systematic cancer survival analyses of alternative splicing at the individual exon resolution have been lacking. Two main challenges exist for survival analyses of mRNA isoform variation and alternative splicing using RNA-seq data. The first challenge is to account for the estimation uncertainty of mRNA isoform ratios inferred from RNA-seq read counts. The statistical confidence of mRNA isoform ratio estimation depends on the RNA-seq read coverage for the events of interest, with larger read coverage leading to a more reliable estimation^14^. Modelling the estimation uncertainty of mRNA isoform ratio is an essential component of RNA-seq analyses of alternative splicing, as shown by various statistical algorithms developed for detecting differential alternative splicing from multi-group RNA-seq data^14^,^26^,^27^,^28^,^29^. The second challenge, which is a general issue in survival analysis, is to properly model the association of mRNA isoform ratio with survival time, while accounting for missing data in survival time because of censoring, that is, patients still alive at the end of the survival study, whose precise survival time would be uncertain. To date, no algorithm has been developed for survival analyses of mRNA isoform variation that accounts for these sources of uncertainty simultaneously.

Here we introduce SURVIV (Survival analysis of mRNA Isoform Variation), a statistical model for identifying mRNA isoform ratios associated with patient survival times in large-scale cancer RNA-seq data sets. SURVIV models the estimation uncertainty of mRNA isoform ratios in RNA-seq data and tests the survival effects of isoform variation in both censored and uncensored survival data. In simulation studies, SURVIV consistently outperforms the conventional Cox regression survival analysis that ignores the measurement uncertainty of mRNA isoform ratio. We used SURVIV to identify alternatively spliced exons whose exon-inclusion levels significantly correlated with the survival times of invasive ductal carcinoma (IDC) patients from the TCGA breast cancer cohort. Survival-associated alternative splicing events are identified in gene pathways associated with apoptosis, oxidative stress and DNA damage repair. Importantly, we show that alternative splicing-based survival predictors outperform gene expression-based survival predictors in the TCGA IDC RNA-seq data set, as well as in TCGA data of five additional cancer types. Moreover, the integration of clinical information, gene expression and alternative splicing profiles leads to the best prediction of survival time.

## Results

### SURVIV statistical model

**Figure 1:**
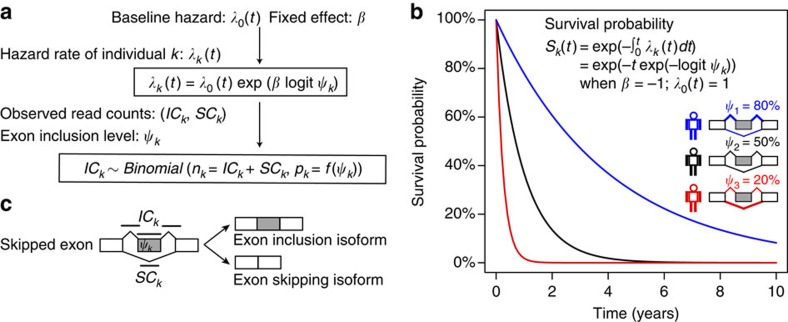
The statistical framework of the SURVIV model. (**a**) For each patient *k*, the patient's hazard rate *λ*_*k*_(*t*) is associated with the baseline hazard rate *λ*_0_(*t*) and this patient's exon-inclusion level *ψ*_*k*_. The association of exon-inclusion level with patient survival is estimated by the survival coefficient *β*. The exon-inclusion level *ψ*_*k*_ is estimated from the read counts for the exon-inclusion isoform *IC*_*k*_ and the exon-skipping isoform *SC*_*k*_. The proportion of the inclusion and skipping reads is adjusted by a normalization function *f* that considers the lengths of the exon-inclusion and -skipping isoforms (see details in Results and Supplementary Methods). (**b**) A hypothetical example to illustrate the association of exon-inclusion level with patient survival probability over time *S*_*k*_(*t*), with the survival coefficient *β*=−1 and a constant baseline hazard rate *λ*_0_(*t*)=1. In this example, patients with higher exon-inclusion levels have lower hazard rates and higher survival probabilities. (**c**) The schematic diagram of an exon-skipping event. The exon-inclusion reads *IC*_*k*_ are the reads from the upstream splice junction, the alternative exon itself and the downstream splice junction. The exon-skipping reads *SC*_*k*_ are the reads from the skipping splice junction that directly connects the upstream exon to the downstream exon.

The statistical model of SURVIV assesses the association between mRNA isoform ratio and patient survival time. While the model is generic for many types of alternative isoform variation, here we use the exon-skipping type of alternative splicing to illustrate the model (Fig. 1a). For each alternative exon involved in exon-skipping, we can use the RNA-seq reads mapping to its exon-inclusion or -skipping isoform to estimate its exon-inclusion level (denoted as *ψ*, or PSI that is Per cent Spliced In^14^). A key feature of SURVIV is that it models the RNA-seq estimation uncertainty of exon-inclusion level as influenced by the sequencing coverage for the alternative splicing event of interest. This is a critical issue in accurate quantitative analyses of mRNA isoform ratio in large-scale RNA-seq data sets^14^,^26^,^27^,^28^,^29^. Therefore, SURVIV contains two major components: the first to model the association of mRNA isoform ratio with patient survival time across all patients, and the second to model the estimation uncertainty of mRNA isoform ratio in each individual patient (Fig. 1a).

Briefly, for any individual exon-skipping event, the first component of SURVIV uses a proportional hazards model to establish the relationship between patient *k*'s exon-inclusion level *ψ*_*k*_ and hazard rate *λ*_*k*_(*t*).


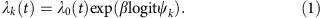


For each exon, the association between the exon-inclusion level and patient survival time is reflected by the survival coefficient *β*. A positive *β* means increased exon inclusion is associated with higher hazard rate and poorer survival, while a negative *β* means increased exon inclusion is associated with lower hazard rate and better survival. *λ*_0_(*t*) is the baseline hazard rate estimated from the survival data of all patients (see Supplementary Methods for the detailed estimation procedure). A particular patient's survival probability over time *S*_*k*_(*t*) can be calculated from the patient-specific hazard rate *λ*_*k*_(*t*) as 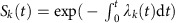. Figure 1b illustrates a simple example with a negative *β*=−1 and a constant baseline hazard rate *λ*_0_(*t*)=1, where higher exon-inclusion levels are associated with lower hazard rates and higher survival probabilities.

The second component of SURVIV models the exon-inclusion level and its estimation uncertainty in individual patient samples. As illustrated in Fig. 1c, the exon-inclusion level *ψ*_*k*_ of a given exon in a particular sample can be estimated by the RNA-seq read count specific to the exon inclusion isoform (*IC*_*k*_) and the exon-skipping isoform (*SC*_*k*_). Other types of alternative splicing and mRNA isoform variation can be similarly modelled by this framework^29^. Given the effective lengths (that is, the number of unique isoform-specific read positions) of the exon-inclusion isoform (*l*_I_) and the exon-skipping isoform (*l*_S_), the exon-inclusion level *ψ*_*k*_ can be estimated as 
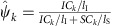
. Assuming that the exon-inclusion read count *IC*_*k*_ follows a binomial distribution with the total read count *n*_*k*_=*IC*_*k*_+*SC*_*k*_, we have:


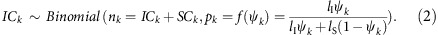


The binomial distribution models the estimation uncertainty of *ψ*_*k*_ as influenced by the total read count *n*_*k*_, in which the parameter *p*_*k*_ represents the proportion of reads from the exon-inclusion isoform, given the exon-inclusion level *ψ*_*k*_ adjusted by a length normalization function *f*(*ψ*_*k*_) based on the effective lengths of the isoforms. The definitions of effective lengths for all basic types of alternative splicing patterns are described in ref. 29.

Distinct from conventional survival analyses in which predictors do not have estimation uncertainty, the predictors in SURVIV are exon-inclusion levels *ψ*_*k*_ estimated from RNA-seq count data, and the confidence of *ψ*_*k*_ estimate for a given exon in a particular sample depends on the RNA-seq read coverage. We use the statistical framework of survival measurement error model^30^ to incorporate the estimation uncertainty of isoform ratio in the proportional hazards model. Using a likelihood ratio test, we test whether the exon-inclusion levels have a significant association with patient survival over the null hypothesis *H*_0_:*β*=0. The false discovery rate (FDR) is estimated using the Benjamini and Hochberg approach^31^. Details of the parameter estimation and likelihood ratio test in SURVIV are described in Supplementary Methods.

### Simulation studies of SURVIV

**Figure 2:**
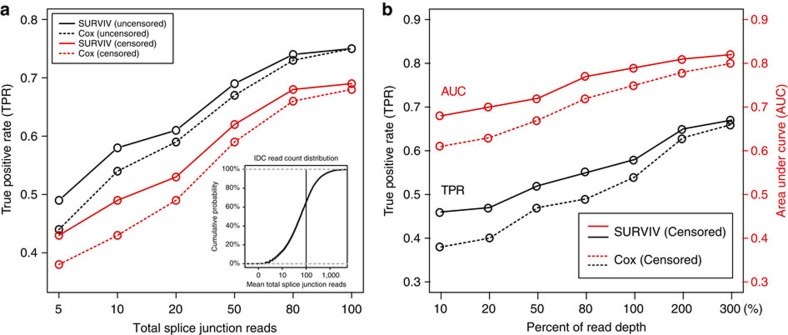
Simulation studies to assess the performance of SURVIV and the importance of modelling the estimation uncertainty of mRNA isoform ratio. We compared our SURVIV model with Cox regression using point estimates of exon-inclusion levels, which does not consider the estimation uncertainty of the mRNA isoform ratio. (**a**) To study the effect of RNA-seq depth, we simulated the mean total splice junction read counts equal to 5, 10, 20, 50, 80 and 100 reads. We generated two sets of simulations with and without data-censoring. For each simulation, the true-positive rate (TPR) at 5% false-positive rate is plotted. The inset figure shows the empirical distribution of the mean total splice junction read counts in the TCGA IDC RNA-seq data (*x* axis in the log10 scale). (**b**) To faithfully represent the read count distribution in a real data set, we performed another simulation with read counts directly sampled from the TCGA IDC data. Sampled read counts were then multiplied by different factors ranging from 10 to 300% to simulate data sets with different RNA-seq read depth. Continuous and dashed lines represent the performance of SURVIV and Cox regression, respectively. Red lines represent the area under curve (AUC) of the ROC curve (TPR versus false-positive rate plot). Black lines represent the TPR at 5% false-positive rate.

We designed a set of simulation studies to compare the performance of SURVIV with a conventional Cox regression survival analysis using the point estimates of exon-inclusion levels. To recapitulate the features of real data sets, we mimicked the parameters of the TCGA IDC breast cancer data wherever possible in generating the simulated data. Specifically, we simulated 20,000 alternative exons in each simulation (similar to the number of exon-skipping events in the TCGA IDC data), with 90% of the exons from the null hypothesis that the exons were not associated with survival time (*β*=0), and the remaining 10% of the exons from the alternative hypothesis that the exons were associated with survival time. The survival coefficient *β* of exons representing the alternative hypothesis was randomly sampled from the top 100 significant exons based on SURVIV analysis of all IDC patients (see Supplementary Data 1). We simulated a data set of 600 individuals, which was close to the sample size of the TCGA IDC data set. To study the effect of RNA-seq coverage, we simulated the total number of exon-inclusion plus exon-skipping splice junction reads (*n*) with mean value (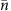) of 5, 10, 20, 50, 80 and 100 (Fig. 2a). The inset figure in Fig. 2a shows the cumulative distribution of the mean splice junction read counts for individual exon-skipping events averaged across all patients in the TCGA IDC RNA-seq data. The read counts for a given exon in individual patients were then simulated using a Gamma–Poisson model with the variance of read counts estimated from the TCGA IDC data. Details about the simulation procedures are described in the Methods section.

Using these simulated data, we compared SURVIV with Cox regression in two settings, without or with censoring of the survival time. In the setting without censoring, the death and survival time of each individual is known. In the setting with censoring, certain individuals are still alive at the end of the survival study. Consequently, these patients have unknown death and survival time. Here, in the simulation with censoring, we assumed that 85% of the patients were still alive at the end of the study, similar to the censoring rate of the TCGA IDC data set. In both settings and with different depths of RNA-seq coverage, SURVIV consistently outperformed Cox regression in the true-positive rate at the same false-positive rate of 5% (Fig. 2a). As expected, we observed a more significant improvement in SURVIV over Cox regression when the RNA-seq read coverage was low (Fig. 2a).

To more faithfully recapitulate the read count distribution in a real cancer RNA-seq data set, we performed another simulation study with read counts directly sampled from the TCGA IDC data. To assess the influence of RNA-seq read depth on the performance of SURVIV and Cox regression, sampled read counts were then multiplied by different factors ranging from 10 to 300% to simulate data sets with different RNA-seq read depths (Fig. 2b). The TCGA IDC data set has an average RNA-seq depth of ∼60 million paired-end reads per patient. Thus, the read depth of these simulated RNA-seq data sets ranged from ∼6 million reads to 180 million reads per patient, representing low-coverage RNA-seq studies designed primarily for gene expression analysis^32^ up to high-coverage RNA-seq studies designed primarily for alternative isoform analysis^29^. At all levels of RNA-seq depth, SURVIV consistently outperformed Cox regression, as reflected by the area under curve of the receiver operating characteristic (ROC) curve as well as the true-positive rate at 5% false-positive rate (Fig. 2b). The improvement of SURVIV over Cox regression was particularly prominent when the read depth was low. For example, at 10% read depth, SURVIV had 7% improvement in area under curve (68% versus 61%) and 8% improvement in the true-positive rate at 5% false-positive rate (46% versus 38%). Collectively, these simulation results suggest that SURVIV achieves a higher accuracy by accounting for the estimation uncertainty of mRNA isoform ratio in RNA-seq data.

### SURVIV analysis of TCGA IDC breast cancer data

**Figure 3:**
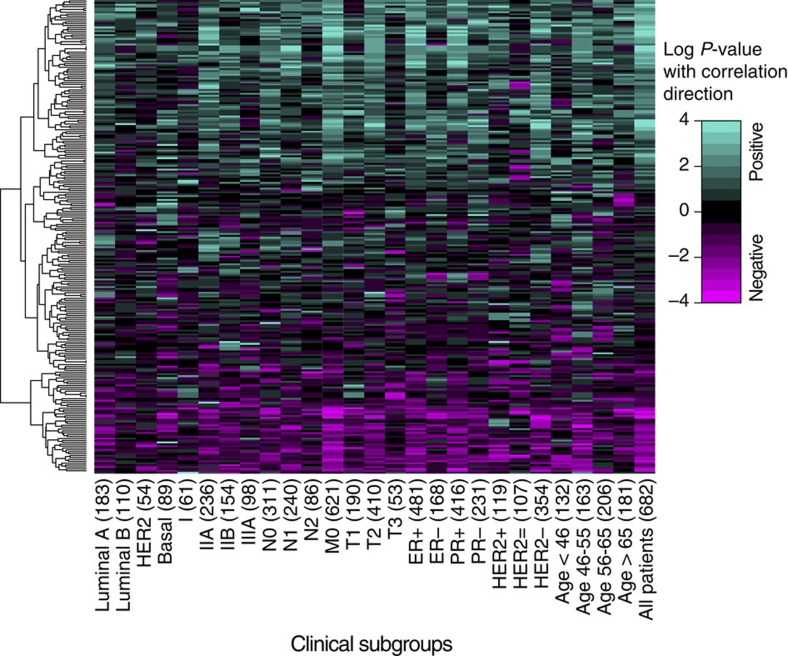
SURVIV analysis of exon-skipping events in the TCGA IDC RNA-seq data set. IDC patients are stratified into multiple clinical subgroups based on clinical parameters including cancer subtype, stage, lymph node status, metastasis, tumour size, oestrogen receptor status, progesterone receptor status, HER2 status and age. Only clinical subgroups with at least 50 patients are included in further analyses. Numbers of patients in the subgroups are indicated next to the names of the subgroups. Shown in the heatmap are the log10 SURVIV *P*-values of the 229 exons associated with patient survival (*P*≤0.01) in at least two subgroups of the same class of clinical parameters. Turquoise colour indicates positive correlation that higher exon-inclusion levels are associated with higher survival probabilities. Magenta colour indicates negative correlation that lower exon-inclusion levels are associated with higher survival probabilities.

**Figure 4:**
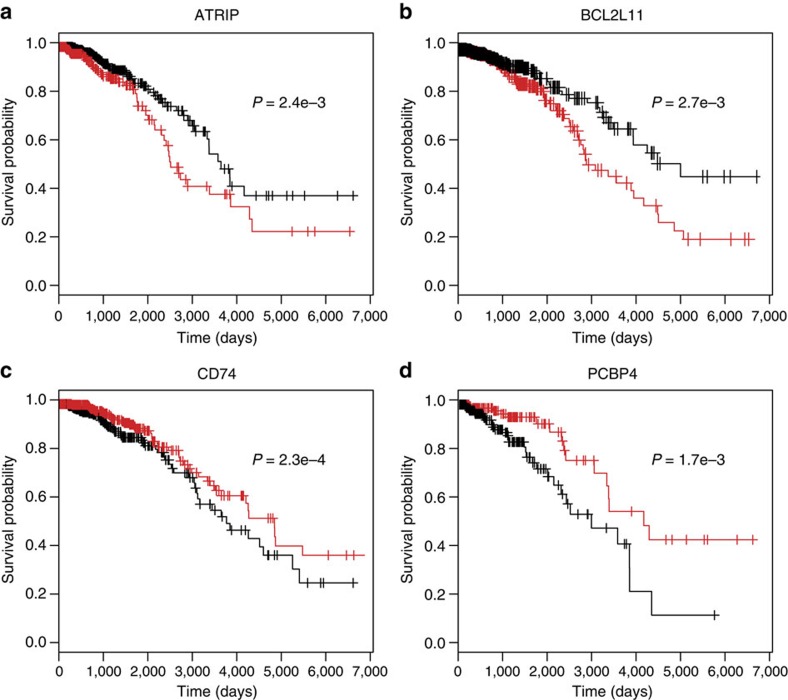
Kaplan–Meier survival plots of IDC patients stratified by two-means clustering of the exon-inclusion levels of four survival-associated alternative splicing events. Clustering was generated for each of the four exons separately. Black lines represent patients with high exon-inclusion levels. Red lines represent patients with low exon-inclusion levels. The *P*-values are from SURVIV analysis of the TCGA IDC RNA-seq data. (**a**) *ATRIP*. (**b**) *BCL2L11*. (**c**) *CD74*. (**d**) *PCBP4*.

To illustrate the practical utility of SURVIV, we used it to analyse the overall survival time of 682 IDC patients from the TCGA breast cancer (BRCA) RNA-seq data set (see Methods for details of the data source and processing pipeline). We chose to analyse IDC because it is the most frequent type of breast cancer^33^, comprising ∼70% of patients in the TCGA breast cancer data set. To control for the effects of significant clinical parameters such as tumour stage and subtype and identify alternative splicing events associated with patient outcomes across multiple molecular and clinical subtypes, we followed the procedure of Croce and colleagues in analysing mRNA and microRNA prognostic signature of IDC^33^ and stratified the patients according to their clinical parameters. We then conducted SURVIV analysis in 26 clinical subgroups with at least 50 patients in each subgroup. We identified 229 exon-skipping events associated with patient survival in multiple clinical subgroups that met the criteria of SURVIV *P*-value≤0.01 in at least two subgroups of the same clinical parameter (cancer subtype, stage, lymph node, metastasis, tumour size, oestrogen receptor status, progesterone receptor status, HER2 status and age as shown in Fig. 3). DAVID (Database for Annotation, Visualization and Integrated Discovery) Gene Ontology analyses^34^ of the 229 alternative splicing events suggest an enrichment of genes in cancer-related functional categories such as intracellular signalling, apoptosis, oxidative stress and response to DNA damage (Supplementary Fig. 1). Table 1 shows a few selected examples of survival-associated alternative splicing events in cancer-related genes. Using two-means clustering of each individual exon's inclusion levels, the 682 IDC patients can be segregated into two subgroups with significantly different survival times as illustrated by the Kaplan–Meier survival plot (Fig. 4). We also carried out hierarchical clustering of IDC patients using 176 survival-associated alternative exons (*P*≤0.01; SURVIV analysis of all IDC patients). Using the exon-inclusion levels of these 176 exons, we clustered IDC patients into three major subgroups, with 95, 194 and 389 patients, respectively. As illustrated by the Kaplan–Meier survival plots, the three subgroups had significantly different survival times (Supplementary Fig. 2).

**Figure 5:**
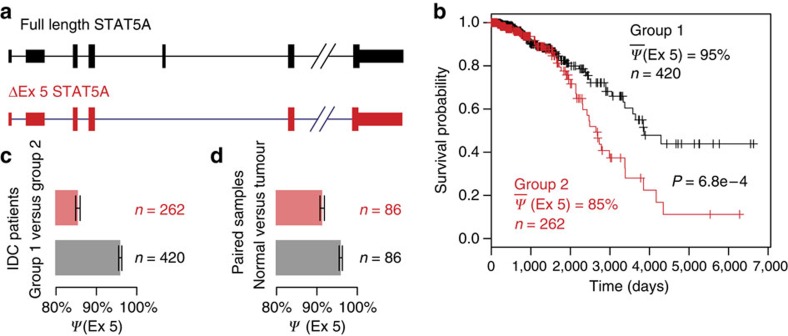
Alternative splicing of *STAT5A* exon 5 is significantly associated with IDC patient survival. (**a**) The gene structure of the *STAT5A* full-length isoform compared to the ΔEx5 isoform skipping the 5th exon. (**b**) Kaplan–Meier survival plot of IDC patients stratified by two-means clustering using exon-inclusion levels of *STAT5A* exon 5. The 420 patients in Group 1 (average exon 5 inclusion level=95%) have significantly higher survival probabilities than the 262 patients in Group 2 (average exon 5 inclusion level=85%) (SURVIV *P*=6.8e−4). (**c**) Exon 5 inclusion levels of IDC patients stratified by two-means clustering using exon 5 inclusion levels. Group 1 has 420 patients with average exon-inclusion level at 95%. Group 2 has 262 patients with average exon-inclusion level at 85%. (**d**) *STAT5A* exon 5 inclusion levels in normal breast tissues versus breast cancer tumour samples. Exon-inclusion levels are extracted from 86 TCGA breast cancer patients with matched normal and tumour samples. Normal breast tissues have average exon 5 inclusion level at 95%, compared to 91% average exon-inclusion level in tumour samples. Error bars represent 95% confidence interval of the mean.

As an example of a survival-associated alternative splicing event with potential functional significance, an exon-skipping event of *STAT5A* (signal transducer and activator of transcription 5a) was found to be significantly associated with IDC patient survival (Fig. 5). STAT5A is a signal transducer and transcriptional activator involved in the programmed cell death^35^. It is activated by a number of cytokines. Once activated, STAT5A enters the nucleus and functions as a transcription factor^36^. The activation of STAT5A is important to both cell growth and differentiation. The alternatively spliced *STAT5A* mRNA isoform lacking exon 5 encodes a protein variant (ΔEx5 isoform; see Fig. 5a) that inhibits the production of p21 and Bax and increases cell number^37^. The STAT5A ΔEx5 protein isoform was found to have only 10% transcriptional regulatory activity as compared with the full-length STAT5A (ref. 37). We found that the inclusion levels of *STAT5A* exon 5 had a significant positive association with IDC patient survival (SURVIV *P*=6.8e−4; Fig. 5b). When we clustered the 682 IDC patients into two subgroups according to the inclusion levels of *STAT5A* exon 5, the first group of 420 patients with higher exon 5 inclusion levels (∼95% on average) had significantly longer survival times as compared with the second group of 262 patients with lower exon 5 inclusion levels (∼85% on average; Fig. 5b,c). We also analysed *STAT5A* exon 5 in a separate set of TCGA breast cancer RNA-seq data with tumour-normal-matched pairs. Consistent with higher exon 5 inclusion levels associated with more favourable patient outcomes, normal breast tissues had 95% exon 5 inclusion levels as compared with 91% exon-inclusion levels in tumour samples (Fig. 5d). This example illustrates that the survival analysis of mRNA isoform variation can identify alternative splicing events with interesting functional implications in cancer development and progression.

### Network of survival-associated alternative splicing events

**Figure 6:**
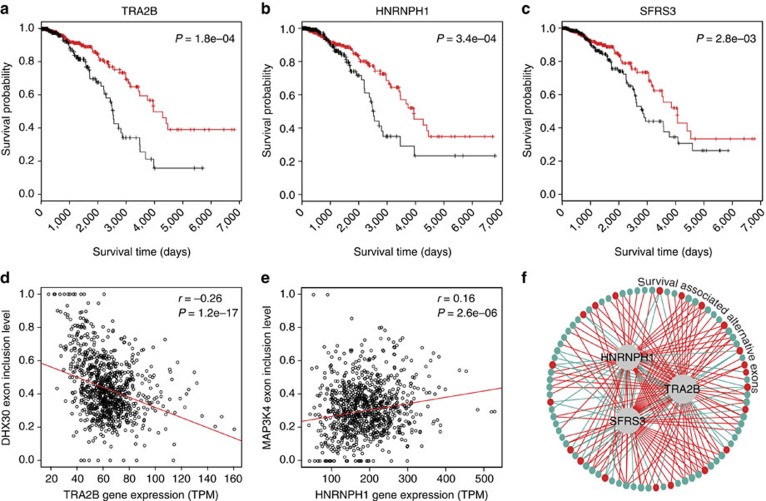
Splicing factor regulatory network of survival-associated alternative splicing events in IDC. (**a**–**c**) Kaplan–Meier survival plots of IDC patients stratified by the gene expression levels of three splicing factors: *TRA2B* (**a**, Cox regression *P*=1.8e−4), *HNRNPH1* (**b**, *P*=3.4e−4) and *SFRS3* (**c**, *P*=2.8e−3). Black lines represent patients with high gene expression levels. Red lines represent patients with low gene expression levels. (**d**) The exon-inclusion levels of a *DHX30* alternative exon are negatively correlated with *TRA2B* gene expression levels (robust correlation coefficient *r*=−0.26, correlation *P=*1.2e−17). (**e**) The exon-inclusion levels of a *MAP3K4* alternative exon are positively correlated with *HNRNPH1* gene expression levels (robust correlation coefficient *r*=0.16, correlation *P=*2.6e−06). (**f**) A splicing co-expression network of the three splicing factors and their correlated survival-associated alternative exons. In total, 84 survival-associated alternative exons are significantly correlated with the three splicing factors. The positive/negative correlation between splicing factors and alternative exons is represented by blue/red lines, respectively. Exons whose inclusion levels are positively/negatively correlated with survival times are represented by blue/red dots, respectively. The size of the splicing factor circles is proportional to the number of correlated exons within the network.

Next, we characterized the splicing-regulatory network of the 229 alternative splicing events associated with IDC patient survival. Specifically, we asked whether a significant fraction of these alternative splicing events were potentially regulated by a few key splicing factors with altered expression in IDC breast cancer tissues. Splicing factors are RNA-binding proteins that recognize *cis*-regulatory elements within the pre-mRNA to influence exon selection and splice site choice. Using gene expression levels calculated from the TCGA IDC RNA-seq data, we identified six splicing factors whose gene expression levels were significantly associated with IDC patient survival time (Cox regression FDR≤1%, Supplementary Data 2). To identify potential exon targets of these six splicing factors among the 229 survival-associated alternative splicing events, we performed a gene–exon co-expression network analysis. Specifically, we used robust regression (R package ‘robust') to test the correlation between the gene expression levels of the splicing factors and the exon-inclusion levels of survival-associated alternative splicing events. Each of the six splicing factors had 41–61 significantly correlated exons among the 229 alternative splicing events (robust regression FDR≤1%). We then investigated whether the correlated exons of a given splicing factor were significantly enriched among the 229 survival-associated alternative splicing events. Among the six splicing factors, three (TRA2B, HNRNPH1 and SFRS3) had significant enrichment in the co-expression network of alternative splicing events (Fisher's exact test FDR≤1%, see detailed data in Supplementary Data 2). As illustrated in Fig. 6a–c, for each of the three splicing factors, the gene expression levels were significantly correlated with patient survival times, with higher gene expression associated with shorter survival time and poorer prognosis. The expression levels of these splicing factors were strongly correlated or anticorrelated with the exon-inclusion levels of survival-associated alternative splicing events in IDC RNA-seq data. For example, DHX30 is a member of the DEAD-box proteins involved in RNA metabolism^38^. Exon 4 of *DHX30* was negatively correlated with splicing factor TRA2B (robust correlation *P*-value=1.2e−17, *r*=−0.26, Fig. 6d). Likewise, exon 17 of *MAP3K4* was positively correlated with splicing factor HNRNPH1 (robust correlation *P*-value=2.6e−06, *r*=0.16, Fig. 6e). MAP3K4 is a component of the mitogen-activated protein kinase pathway, which plays critical roles in cancer development^39^. Collectively, these three splicing factors were significantly correlated with 37% (84 out of 229) of the survival-associated alternative splicing events, thus placing them at the ‘hubs' of the survival-associated splicing regulatory network in IDC (Fig. 6f).

A total of 165 significant correlations existed among the 84 survival-associated alternative exons and the three splicing factors in this network (Fig. 6f). As the expression levels of all three splicing factors were negatively associated with survival, exons positively associated with these splicing factors (blue lines) should have negative association between exon-inclusion levels and patient survival times (red dots), while exons negatively associated with these splicing factors (red lines) should have positive association between exon-inclusion levels and patient survival times (blue dots). The majority of the 165 significant correlations had the expected correlation directions between the splicing factor expression level and exon-inclusion level (135/165=82%, two-sided binomial test *P*<2.2e−16).

Our combined survival and co-expression network analysis identified splicing factors known to be dysregulated in breast cancer. For example, TRA2B is known to have elevated expression in breast cancers compared with normal breast tissues, and it regulates the alternative splicing of a number of genes with key functions in cancer development^40^,^41^,^42^,^43^. The TRA2B target genes encode key proteins involved in cell proliferation, survival and migration^44^. The splicing factor HNRNPH1 was reported to inhibit apoptosis in cancer cells partially by regulating the A-Raf kinase in the mitogen-activated protein kinase pathway^45^.

### Alternative splicing predictors of cancer patient survival

**Figure 7:**
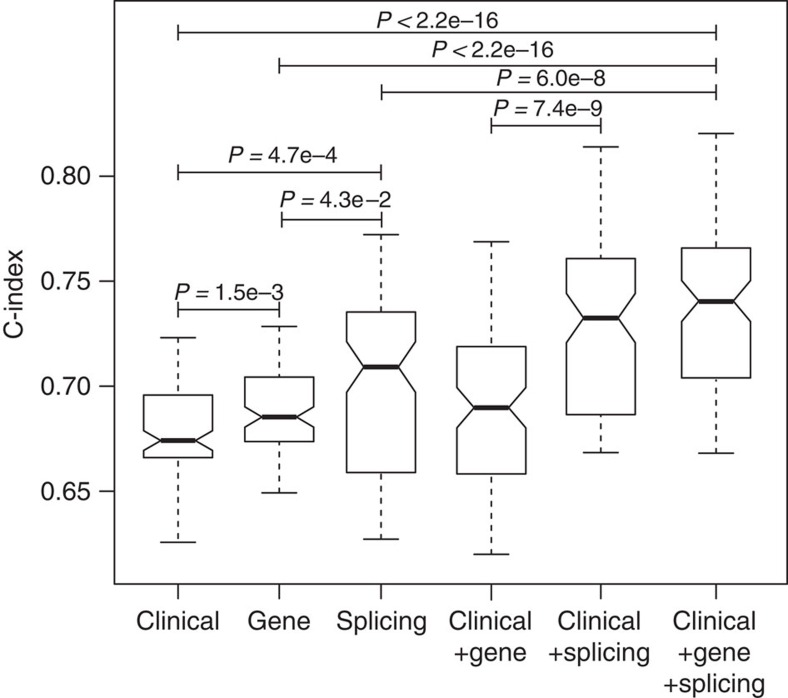
Cross-validation of different classes of IDC survival predictors measured by the C-index. A C-index of 1 indicates perfect prediction accuracy and a C-index of 0.5 indicates random guess. The plots indicate the distribution of C-indexes from 100 rounds of cross-validation. The centre value of the box plot is the median C-index from 100 rounds of cross-validation. The notch represents the 95% confidence interval of the median. The box represents the 25 and 75% quantiles. The whiskers extended out from the box represent the 5 and 95% quantiles. Two-sided Wilcoxon test was used to compare different survival predictors. The different classes of predictors are: (a) clinical information (median C-index 0.67). (b) Gene expression (median C-index 0.68). (c) Alternative splicing (median C-index 0.71). (d) Clinical information+gene expression (median C-index 0.69). (e) Clinical information+alternative splicing (median C-index 0.73). (f) Clinical information+gene expression+alternative splicing (median C-index 0.74). Note that ‘Gene' refers to ‘Gene-level expression' in these plots.

Finally, we investigated the prognostic value of alternative splicing as predictors of IDC patient survival. Recent work by the TCGA consortium evaluated the clinical utilities of diverse molecular data (for example, copy number variation, DNA methylation, mRNA and microRNA expression and protein expression) as well as clinical parameters in predicting cancer patient survival^16^. Here we asked whether the use of alternative splicing information can lead to comparable or improved accuracy in predicting patient survival. To assess the clinical utility of alternative splicing-based survival predictors in an unbiased manner, we performed a two-fold Monte Carlo cross-validation analysis using the TCGA IDC RNA-seq data (see details in Methods). Briefly, in each round of cross-validation, the IDC patients were randomly split into a training group of 50% patients and a separate testing group of the remaining 50% patients. We used SURVIV to identify survival-associated alternative splicing events in the training group with a loose *P*-value threshold of ≤0.01. Then, a penalized Cox regression of L1 penalty was used to build the predictive model combining the individual alternative splicing events identified by SURVIV. We then applied the predictive model built from the training group to predict the survival outcome in the testing group. The cross-validation was repeated for 100 times. The discriminatory power of the survival predictors was measured by the C-index^46^. A C-index of 1 indicates perfect prediction accuracy and a C-index of 0.5 indicates random guess. Besides alternative splicing-based predictors, we also built other predictive models based on clinical parameters or gene expression profiles. As shown in Fig. 7, both gene expression and alternative splicing-based predictors performed better than clinical parameters alone (gene versus clinical *P*=1.5e−3; splicing versus clinical *P*=4.7e−4; two-sided Wilcoxon test). We also observed that alternative splicing predictors alone performed better than gene expression predictors (splicing versus gene *P*=4.3e−2, two-sided Wilcoxon test). Adding clinical parameters to the predictive model collectively improved the performance of both gene expression and alternative splicing-based predictors, while the combined model of alternative splicing plus clinical parameters outperformed the combined model of gene expression plus clinical parameters (*P*=7.4e−9, two-sided Wilcoxon test). We also evaluated the combined effect of incorporating clinical, gene expression and alternative splicing data. A predictive model incorporating all three types of information performed significantly better than any one type of predictors alone (clinical+gene+splicing versus clinical *P<*2.2e−16; versus gene *P*<2.2e−16; versus splicing *P*=6.0e−8, two-sided Wilcoxon test). The improved prediction accuracy was not caused by over-fitting since the training and testing data were completely separated during cross-validation. This result illustrates the potential value of utilizing alternative splicing information for improved prognosis of cancer patients.

Next, we carried out the SURVIV analysis in five additional cancer types in TCGA, including GBM (glioblastoma multiforme), KIRC (kidney renal clear cell carcinoma), LGG (lower grade glioma), LUSC (lung squamous cell carcinoma) and OV (ovarian serous cystadenocarcinoma). As expected, the number of significant events at different FDR or *P*-value significance cutoffs varied across cancer types, with LGG having the strongest survival-associated alternative splicing signals with 660 significant exon-skipping events at FDR≤5% (Supplementary Data 3 and 4). Strikingly, regardless of the number of significant events, alternative splicing-based survival predictors outperformed gene expression-based survival predictors across all cancer types (Supplementary Fig. 3), consistent with our initial observation on the IDC data set.

## Discussion

Alternative processing and modification of mRNA, such as alternative splicing, allow cells to generate a large number of mRNA and protein isoforms with diverse regulatory and functional properties. The plasticity of alternative splicing is often exploited by cancer cells to produce isoform switches that promote cancer cell survival, proliferation and metastasis^7^,^8^. The widespread use of RNA-seq in cancer transcriptome studies^15^,^47^,^48^ has provided the opportunity to comprehensively elucidate the landscape of alternative splicing in cancer tissues. While existing studies of alternative splicing in large-scale cancer transcriptome data largely focused on the comparison of splicing patterns between cancer and normal tissues or between different subtypes of cancer^18^,^21^,^49^, additional computational tools are needed to characterize the clinical relevance of alternative splicing using massive RNA-seq data sets, including the association of alternative splicing with phenotypes and patient outcomes.

We have developed SURVIV, a novel statistical model for survival analysis of alternative isoform variation using cancer RNA-seq data. SURVIV uses a survival measurement error model to simultaneously model the estimation uncertainty of mRNA isoform ratio in individual patients and the association of mRNA isoform ratio with survival time across patients. Compared with the conventional Cox regression model that uses each patient's mRNA isoform ratio as a point estimate, SURVIV achieves a higher accuracy as indicated by simulation studies under a variety of settings. Of note, we observed a particularly marked improvement of SURVIV over Cox regression for low- and moderate-depth RNA-seq data (Fig. 2b). This has important practical value because many clinical RNA-seq data sets have large sample size but relatively modest sequencing depth.

Using the TCGA IDC breast cancer RNA-seq data of 682 patients, SURVIV identified 229 alternative splicing events associated with patient survival time, which met the criteria of SURVIV *P*-values≤0.01 in multiple clinical subgroups. While the statistical threshold seemed loose, several lines of evidence suggest the functional and clinical relevance of these survival-associated alternative splicing events. These alternative splicing events were frequently identified and enriched in the gene functional groups important for cancer development and progression, including apoptosis, DNA damage response and oxidative stress. While some of these events may simply reflect correlation but not causal effect on cancer patient survival, other events may play an active role in regulating cancer cell phenotypes. For example, a survival-associated alternative splicing event involving exon 5 of *STAT5A* is known to regulate the activity of this transcription factor with important roles in epithelial cell growth and apoptosis^37^. Using a co-expression network analysis of splicing factor to exon correlation across all patients, we identified three splicing factors (TRA2B, HNRNPH1 and SFRS3) as potential hubs of the survival-associated alternative splicing network of IDC. The expression levels of all three splicing factors were negatively associated with patient survival times (Fig. 6a–c), and both TRA2B and HNRNPH1 were previously reported to have an impact on cancer-related molecular pathways^40^,^41^,^42^,^43^,^44^,^45^. Finally, despite the limited power in detecting individual events, we show that the survival-associated alternative splicing events can be used to construct a predictor for patient survival, with an accuracy higher than predictors based on clinical parameters or gene expression profiles (Fig. 7). This further demonstrates the potential biological relevance and clinical utility of the identified alternative splicing events.

We performed cross-validation analyses to evaluate and compare the prognostic value of alternative splicing, gene expression and clinical information for predicting patient survival, either independently or in combination. As expected, the combined use of all three types of information led to the best prediction accuracy. Because we used penalized regression to build the prediction model, combining information from multiple layers of data did not necessarily increase the number of predictors in the model. The perhaps more surprising and intriguing result is that alternative splicing-based predictors appear to outperform gene expression-based predictors when used alone and when either type of data was combined with clinical information (Fig. 7). We observed the same trend in five additional cancer types (Supplementary Fig. 3). We note that this finding was consistent with a previous report that cancer subtype classification based on splicing isoform expression performed better than gene expression-based classification^25^. While this trend seems counterintuitive because accurate estimation of gene expression requires much lower RNA-seq depth than accurate estimation of alternative splicing^29^, one possible explanation may be the inherent characteristic of isoform ratio data. By definition, mRNA isoform ratio is estimated as the ratio of multiple mRNA isoforms from a single gene. Therefore, mRNA isoform ratio data have a ‘built-in' internal control that could be more robust against certain artefacts and confounding issues that influence gene expression estimates across large clinical RNA-seq data sets, such as poor sample quality and RNA degradation^12^. Regardless of the reasons, our data call for further studies to fully explore the utility of mRNA isoform ratio data for various clinical research applications.

The SURVIV source code is available for download at https://github.com/Xinglab/SURVIV. SURVIV is a general statistical model for survival analysis of mRNA isoform ratio using RNA-seq data. The current statistical framework of SURVIV is applicable to RNA-seq based count data for all basic types of alternative splicing patterns involving two isoform choices from an alternatively spliced region, such as exon-skipping, alternative 5′ splice sites, alternative 3′ splice sites, mutually exclusive exons and retained introns, as well as other forms of alternative isoform variation such as RNA editing. With the rapid accumulation of clinical RNA-seq data sets, SURVIV will be a useful tool for elucidating the clinical relevance and potential functional significance of alternative isoform variation in cancer and other diseases.

## Methods

### Simulation studies of SURVIV

We designed simulation studies to compare the performance of SURVIV with the conventional Cox regression survival analysis using point estimates of mRNA isoform ratios. We mimicked the characteristics of the TCGA IDC breast cancer RNA-seq data wherever possible in the simulations. For each simulation, we simulated a data set of 600 individuals, which was similar to the sample size of the TCGA IDC data. To study the effect of RNA-seq depth, for individual alternative splicing events we simulated the total number of splice junction reads (*n*) with the mean value (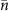) equal to 5, 10, 20, 50, 80 or 100, respectively. We simulated 20,000 exons at each RNA-seq depth, with 10% of the exons from the alternative hypothesis that the exons were associated with survival time. The survival coefficient *β* of individual exons was randomly sampled from the top 100 significant exons in the TCGA IDC data set. The remaining 90% of the exons were from the null hypothesis that the exons were not associated with survival time (*β*=0).

For each exon, we then simulated the total number of splice junction reads and exon-inclusion levels across the 600 individuals. To model the variation in the total splice junction counts across individuals, we used a Gamma–Poisson model to simulate the total number of splice junction reads of each individual *k*. First, we generated the mean of Poisson distribution (*λ*_*k*_) for each individual *k* as: 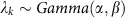, where the shape parameter 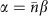 and the rate parameter *β*=0.05, which produced a read count variance similar to the variance in the TCGA IDC data. Then, we generated the total number of splice junction reads (*n*_*k*_) for each individual *k* as: 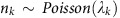. After that, we simulated the mean exon-inclusion level (*ψ*) according to a distribution Beta(0.51,0.36) that fitted the observed distribution of all alternative exons from the TCGA IDC data set. Similarly, the variance of the exon-inclusion levels (*σ*) was simulated according to a distribution *Gamma*(1.31,15.04) that fitted the observed variance of all alternative exons from the TCGA IDC data set. Each individual's exon-inclusion level (*ψ*_*k*_) was simulated as: 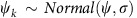. The inclusion splice junction counts were generated as: 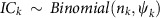. We also performed a second set of simulation, in which we directly sampled the read counts from the read counts of the exon-skipping events in the TCGA IDC data. Sampled read counts were then multiplied by different factors (ranging from 10 to 300%) to simulate data sets with different RNA-seq read depths.

We simulated the patient survival time based on the proportional hazards model, in which the individual exon-inclusion level *ψ*_*k*_ was used as the covariate. We used the Nelson–Aalen estimator^50^ of the baseline hazard function from the TCGA IDC data set as the baseline hazard function of the proportional hazards model. In the simulation with censored data, the censored individuals were randomly selected from the 85% of the individuals (note IDC patients had 89% censoring rate). The censor time was randomly sampled from 10 to 90% of the actual death time.

### TCGA RNA-seq data

We obtained the RNA-seq read counts and clinical parameters of 682 IDC patients from TCGA breast cancer (BRCA) cohort (TCGA dbGaP Accession ID phs000178). The RNA-seq reads were mapped to the human genome and transcriptome by the TCGA consortium using Burrows-Wheeler Aligner (BWA) with default settings^15^. The TCGA consortium provided the numbers of RNA-seq read counts on splice junctions as part of the Level 3 RNA-seq data available at the TCGA data portal (https://tcga-data.nci.nih.gov/tcga/). For each exon-skipping event defined in the Ensembl annotations (release #75), we identified the read counts for both exon-inclusion and -skipping splice junctions from the Level 3 data. The IDC patient list is provided in Supplementary Data 5. The same procedure was used to obtain and process TCGA RNA-seq data and clinical parameters of five additional cancer types, including GBM, KIRC, LGG, LUSC and OV.

### Cross-validation of survival predictors

We evaluated and compared the survival prediction using clinical information, gene expression and alternative splicing. We used two-fold cross-validation to split the IDC patients into separate training and testing groups. The even split of the IDC patients ensured that there were enough death events in both training and testing groups for accurate model training and testing. The cross-validation was repeated for 100 times. Each time, half of the IDC patients were randomly selected as the training group and the other half were selected as the testing group. Using the training group, we built a series of survival prediction models based on clinical information, gene expression or alternative splicing predictors as well as the combination of these predictors. The clinical parameters used in the Monte Carlo cross-validation were identical to those used for the initial SURVIV analysis of 26 clinical subgroups (Fig. 3), which included cancer subtype, stage, lymph node status, metastasis, tumour size, oestrogen receptor status, progesterone receptor status, HER2 status and age. First, we used univariate regression (*P*≤0.01) to select potential survival predictors associated with patient survival time. We used Cox regression for the univariate analysis of clinical information and gene expression. We used SURVIV for the univariate analysis of alternative splicing. Then, starting from the predictors selected from univariate regression, we used a L1-penalized Cox regression model^51^ to select a set of predictors in the multivariate prediction model. Owing to the different numerical scales of gene expression and alternative splicing predictors, when we combined these two types of predictors in the penalized regression, a *z*-score normalization was used to scale the gene expression and alternative splicing predictors in the penalized regression. After the model was built using the training group, we used the remaining patients from the testing group to test the model. The prediction accuracy of the model was measured by the C-index^46^. A C-index of 1 indicates perfect prediction accuracy and a C-index of 0.5 indicates random guess.

## Additional information

**How to cite this article:** Shen, S. *et al*. SURVIV for survival analysis of mRNA isoform variation. *Nat. Commun.* 7:11548 doi: 10.1038/ncomms11548 (2016).

## Supplementary Material

Supplementary Information Supplementary Figures 1-3, Supplementary Methods and Supplementary References

Supplementary Data 1 List of 229 survival associated alternative splicing events in the TCGA IDC data. From column G, the table contains the log10 SURVIV P-values for each clinical subgroup. The positive or negative signs in front of the SURVIV P-values indicate the direction of the association. Positive signs indicate that higher exon inclusion levels are associated with better survival probabilities. Negative signs indicate that lower exon inclusion levels are associated with better survival probabilities.

Supplementary Data 2 List of 6 splicing factors whose gene expression levels are significantly associated with IDC patient survival. In the enrichment analysis, all other alternatively spliced exons from genes containing survival associated alternative splicing events were used as the background control, so that the background exons and the survival associated exons were from the same set of genes and did not have systematic differences in overall gene expression levels and RNA-seq coverage.

Supplementary Data 3 Numbers of significant survival associated alternative exons in six TCGA cancer types at various FDR/P-value significance cutoffs.

Supplementary Data 4 Full lists of survival associated alternative splicing events at nominal P-value cutoff of 0.01 for six cancer types.

Supplementary Data 5 List of IDC patients from TCGA.

## Figures and Tables

**Table 1:** A selected list of exon-skipping events associated with IDC patient survival.

Gene symbol	Ensembl transcript ID (exon number)	SURVIV *P*-value	Gene function
ATRIP	ENST00000320211 (11)	2.4e−3	Encodes a critical component of the DNA damage checkpoint^52^.
BCL2L11	ENST00000308659 (3)	2.7e−3	Induces apoptosis and anoikis^9^,^53^.
CD74	ENST00000518757 (7)	2.3e−4	Regulates antigen presentation for immune response^54^.
PCBP4	ENST00000484633 (2)	1.7e−3	Induced by p53; suppresses cell proliferation^55^,^56^.
RAD17	ENST00000354312 (3)	1.1e−3	Required for cell cycle arrest and DNA damage repair^57^.
TLR5	ENST00000342210 (2)	1.3e−4	Activates innate immune response^58^.

IDC, invasive ductal carcinoma; SURVIV, Survival analysis of mRNA Isoform Variation.

## References

[b1] Nilsen T. W. & Graveley B. R. Expansion of the eukaryotic proteome by alternative splicing. Nature 463, 457–463 (2010). 20110989 10.1038/nature08909 PMC3443858

[b2] Wang E. T. . Alternative isoform regulation in human tissue transcriptomes. Nature 456, 470–476 (2008). 18978772 10.1038/nature07509 PMC2593745

[b3] Pan Q., Shai O., Lee L. J., Frey B. J. & Blencowe B. J. Deep surveying of alternative splicing complexity in the human transcriptome by high-throughput sequencing. Nat. Genet. 40, 1413–1415 (2008). 18978789 10.1038/ng.259

[b4] Venables J. P. Aberrant and alternative splicing in cancer. Cancer Res. 64, 7647–7654 (2004). 15520162 10.1158/0008-5472.CAN-04-1910

[b5] Kim E., Goren A. & Ast G. Insights into the connection between cancer and alternative splicing. Trends Genet. 24, 7–10 (2008). 18054115 10.1016/j.tig.2007.10.001

[b6] Chen J. & Weiss W. A. Alternative splicing in cancer: implications for biology and therapy. Oncogene 34, 1–14 (2015). 24441040 10.1038/onc.2013.570

[b7] Oltean S. & Bates D. O. Hallmarks of alternative splicing in cancer. Oncogene 33, 5311–5318 (2014). 24336324 10.1038/onc.2013.533

[b8] Liu S. & Cheng C. Alternative RNA splicing and cancer. Wiley Interdiscip. Rev. RNA 4, 547–566 (2013). 23765697 10.1002/wrna.1178 PMC4426271

[b9] Moore M. J., Wang Q., Kennedy C. J. & Silver P. A. An alternative splicing network links cell-cycle control to apoptosis. Cell 142, 625–636 (2010). 20705336 10.1016/j.cell.2010.07.019 PMC2924962

[b10] Yang W. & Lu Z. Regulation and function of pyruvate kinase M2 in cancer. Cancer Lett. 339, 153–158 (2013). 23791887 10.1016/j.canlet.2013.06.008 PMC3950276

[b11] De Craene B. & Berx G. Regulatory networks defining EMT during cancer initiation and progression. Nat. Rev. Cancer 13, 97–110 (2013). 23344542 10.1038/nrc3447

[b12] Warzecha C. C. . An ESRP-regulated splicing programme is abrogated during the epithelial-mesenchymal transition. EMBO J. 29, 3286–3300 (2010). 20711167 10.1038/emboj.2010.195 PMC2957203

[b13] Griffith M. . Alternative expression analysis by RNA sequencing. Nat. Methods 7, 843–847 (2010). 20835245 10.1038/nmeth.1503

[b14] Katz Y., Wang E. T., Airoldi E. M. & Burge C. B. Analysis and design of RNA sequencing experiments for identifying isoform regulation. Nat. Methods 7, 1009–1015 (2010). 21057496 10.1038/nmeth.1528 PMC3037023

[b15] TCGA. Comprehensive molecular portraits of human breast tumours. Nature 490, 61–70 (2012). 23000897 10.1038/nature11412 PMC3465532

[b16] Yuan Y. . Assessing the clinical utility of cancer genomic and proteomic data across tumor types. Nat. Biotechnol. 32, 644–652 (2014). 24952901 10.1038/nbt.2940 PMC4102885

[b17] Ludwig J. A. & Weinstein J. N. Biomarkers in cancer staging, prognosis and treatment selection. Nat. Rev. Cancer 5, 845–856 (2005). 16239904 10.1038/nrc1739

[b18] Sebestyen E., Zawisza M. & Eyras E. Detection of recurrent alternative splicing switches in tumor samples reveals novel signatures of cancer. Nucleic Acids Res. 43, 1345–1356 (2015). 25578962 10.1093/nar/gku1392 PMC4330360

[b19] Dargahi D. . A pan-cancer analysis of alternative splicing events reveals novel tumor-associated splice variants of matriptase. Cancer Inform. 13, 167–177 (2014). 25506199 10.4137/CIN.S19435 PMC4259500

[b20] Dorman S. N., Viner C. & Rogan P. K. Splicing mutation analysis reveals previously unrecognized pathways in lymph node-invasive breast cancer. Sci. Rep. 4, 7063 (2014). 25394353 10.1038/srep07063 PMC4231324

[b21] Tsai Y. S., Dominguez D., Gomez S. M. & Wang Z. Transcriptome-wide identification and study of cancer-specific splicing events across multiple tumors. Oncotarget 6, 6825–6839 (2015). 25749525 10.18632/oncotarget.3145 PMC4466652

[b22] Dvinge H. & Bradley R. K. Widespread intron retention diversifies most cancer transcriptomes. Genome Med. 7, 45 (2015). 26113877 10.1186/s13073-015-0168-9 PMC4480902

[b23] Danan-Gotthold M. . Identification of recurrent regulated alternative splicing events across human solid tumors. Nucleic Acids Res. 43, 5130–5144 (2015). 25908786 10.1093/nar/gkv210 PMC4446417

[b24] Suo C. . Integration of somatic mutation, expression and functional data reveals potential driver genes predictive of breast cancer survival. Bioinformatics 31, 2607–2613 (2015). 25810432 10.1093/bioinformatics/btv164

[b25] Pal S. . Isoform-level gene signature improves prognostic stratification and accurately classifies glioblastoma subtypes. Nucleic Acids Res. 42, e64 (2014). 24503249 10.1093/nar/gku121 PMC4005667

[b26] Shen S. . MATS: a Bayesian framework for flexible detection of differential alternative splicing from RNA-Seq data. Nucleic Acids Res. 40, e61 (2012). 22266656 10.1093/nar/gkr1291 PMC3333886

[b27] Trapnell C. . Differential analysis of gene regulation at transcript resolution with RNA-seq. Nat. Biotechnol. 31, 46–53 (2013). 23222703 10.1038/nbt.2450 PMC3869392

[b28] Zhao K., Lu Z. X., Park J. W., Zhou Q. & Xing Y. GLiMMPS: robust statistical model for regulatory variation of alternative splicing using RNA-seq data. Genome Biol. 14, R74 (2013). 23876401 10.1186/gb-2013-14-7-r74 PMC4054007

[b29] Shen S. . rMATS: robust and flexible detection of differential alternative splicing from replicate RNA-Seq data. Proc. Natl Acad. Sci. USA 111, E5593–E5601 (2014). 25480548 10.1073/pnas.1419161111 PMC4280593

[b30] Prentice R. L. Covariate measurement errors and parameter-estimation in a failure time regression-model. Biometrika 69, 331–342 (1982).

[b31] Benjamini Y. & Hochberg Y. Controlling the false discovery rate: a practical and powerful approach to multiple testing. J. R. Stat. Soc. B Methodol. 57, 289–300 (1995).

[b32] Liu Y., Zhou J. & White K. P. RNA-seq differential expression studies: more sequence or more replication? Bioinformatics 30, 301–304 (2014). 24319002 10.1093/bioinformatics/btt688 PMC3904521

[b33] Volinia S. & Croce C. M. Prognostic microRNA/mRNA signature from the integrated analysis of patients with invasive breast cancer. Proc. Natl Acad. Sci. USA 110, 7413–7417 (2013). 23589849 10.1073/pnas.1304977110 PMC3645522

[b34] Huang D. W., Sherman B. T. & Lempicki R. A. Systematic and integrative analysis of large gene lists using DAVID bioinformatics resources. Nat. Protoc. 4, 44–57 (2009). 19131956 10.1038/nprot.2008.211

[b35] Nosaka T. . STAT5 as a molecular regulator of proliferation, differentiation and apoptosis in hematopoietic cells. EMBO J. 18, 4754–4765 (1999). 10469654 10.1093/emboj/18.17.4754 PMC1171548

[b36] Teglund S. . Stat5a and Stat5b proteins have essential and nonessential, or redundant, roles in cytokine responses. Cell 93, 841–850 (1998). 9630227 10.1016/s0092-8674(00)81444-0

[b37] Tan D. . An N-terminal splice variant of human Stat5a that interacts with different transcription factors is the dominant form expressed in invasive ductal carcinoma. Cancer Lett. 346, 148–157 (2014). 24384092 10.1016/j.canlet.2013.12.030 PMC3959904

[b38] Rocak S. & Linder P. DEAD-box proteins: the driving forces behind RNA metabolism. Nat. Rev. Mol. Cell Biol. 5, 232–241 (2004). 14991003 10.1038/nrm1335

[b39] Wagner E. F. & Nebreda A. R. Signal integration by JNK and p38 MAPK pathways in cancer development. Nat. Rev. Cancer 9, 537–549 (2009). 19629069 10.1038/nrc2694

[b40] Best A. . Human Tra2 proteins jointly control a CHEK1 splicing switch among alternative and constitutive target exons. Nat. Commun. 5, 4760 (2014). 25208576 10.1038/ncomms5760 PMC4175592

[b41] Grellscheid S. . Identification of evolutionarily conserved exons as regulated targets for the splicing activator tra2beta in development. PLoS Genet. 7, e1002390 (2011). 22194695 10.1371/journal.pgen.1002390 PMC3240583

[b42] Watermann D. O. . Splicing factor Tra2-beta1 is specifically induced in breast cancer and regulates alternative splicing of the CD44 gene. Cancer Res. 66, 4774–4780 (2006). 16651431 10.1158/0008-5472.CAN-04-3294

[b43] Venables J. P. . Up-regulation of the ubiquitous alternative splicing factor Tra2beta causes inclusion of a germ cell-specific exon. Hum. Mol. Genet. 14, 2289–2303 (2005). 16000324 10.1093/hmg/ddi233

[b44] Best A. . Expression of Tra2 beta in cancer cells as a potential contributory factor to neoplasia and metastasis. Int. J. Cell Biol. 2013, 843781 (2013). 23935626 10.1155/2013/843781 PMC3723085

[b45] Rauch J. . Heterogeneous nuclear ribonucleoprotein H blocks MST2-mediated apoptosis in cancer cells by regulating A-Raf transcription. Cancer Res. 70, 1679–1688 (2010). 20145135 10.1158/0008-5472.CAN-09-2740 PMC2880479

[b46] Pencina M. J. & D'Agostino R. B. Overall C as a measure of discrimination in survival analysis: model specific population value and confidence interval estimation. Stat. Med. 23, 2109–2123 (2004). 15211606 10.1002/sim.1802

[b47] TCGA. Comprehensive molecular characterization of clear cell renal cell carcinoma. Nature 499, 43–49 (2013). 23792563 10.1038/nature12222 PMC3771322

[b48] TCGA. Comprehensive genomic characterization of squamous cell lung cancers. Nature 489, 519–525 (2012). 22960745 10.1038/nature11404 PMC3466113

[b49] Zhao Q. . Tumor-specific isoform switch of the fibroblast growth factor receptor 2 underlies the mesenchymal and malignant phenotypes of clear cell renal cell carcinomas. Clin. Cancer Res. 19, 2460–2472 (2013). 23444225 10.1158/1078-0432.CCR-12-3708 PMC3644028

[b50] Aalen O. Nonparametric inference for a family of counting processes. Ann. Stat. 701–726 (1978).

[b51] Goeman J. J. L1 penalized estimation in the Cox proportional hazards model. Biom. J. 52, 70–84 (2010). 19937997 10.1002/bimj.200900028

[b52] Zou L. & Elledge S. J. Sensing DNA damage through ATRIP recognition of RPA-ssDNA complexes. Science 300, 1542–1548 (2003). 12791985 10.1126/science.1083430

[b53] O'Connor L. . Bim: a novel member of the Bcl-2 family that promotes apoptosis. EMBO J. 17, 384–395 (1998). 9430630 10.1093/emboj/17.2.384 PMC1170389

[b54] Stein R. . CD74: a new candidate target for the immunotherapy of B-cell neoplasms. Clin. Cancer Res. 13, 5556s–5563s (2007). 17875789 10.1158/1078-0432.CCR-07-1167

[b55] Castano Z., Vergara-Irigaray N., Pajares M. J., Montuenga L. M. & Pio R. Expression of alpha CP-4 inhibits cell cycle progression and suppresses tumorigenicity of lung cancer cells. Int. J. Cancer 122, 1512–1520 (2008). 17973258 10.1002/ijc.23236

[b56] Zhu J. & Chen X. MCG10, a novel p53 target gene that encodes a KH domain RNA-binding protein, is capable of inducing apoptosis and cell cycle arrest in G(2)-M. Mol. Cell Biol. 20, 5602–5618 (2000). 10891498 10.1128/mcb.20.15.5602-5618.2000 PMC86022

[b57] Bao S. . ATR/ATM-mediated phosphorylation of human Rad17 is required for genotoxic stress responses. Nature 411, 969–974 (2001). 11418864 10.1038/35082110

[b58] Hayashi F. . The innate immune response to bacterial flagellin is mediated by Toll-like receptor 5. Nature 410, 1099–1103 (2001). 11323673 10.1038/35074106

