# Supplementary material for: SURVIV for survival analysis of mRNA isoform variation
Source: Nat Commun. 2016 Jun 9;7:11548. doi: 10.1038/ncomms11548 (PMC4906168; doi:10.1038/ncomms11548)
Supplement: Supplementary Information — Supplementary Figures 1-3, Supplementary Methods and Supplementary References [file ncomms11548-s1.pdf]

Supplementary Figures

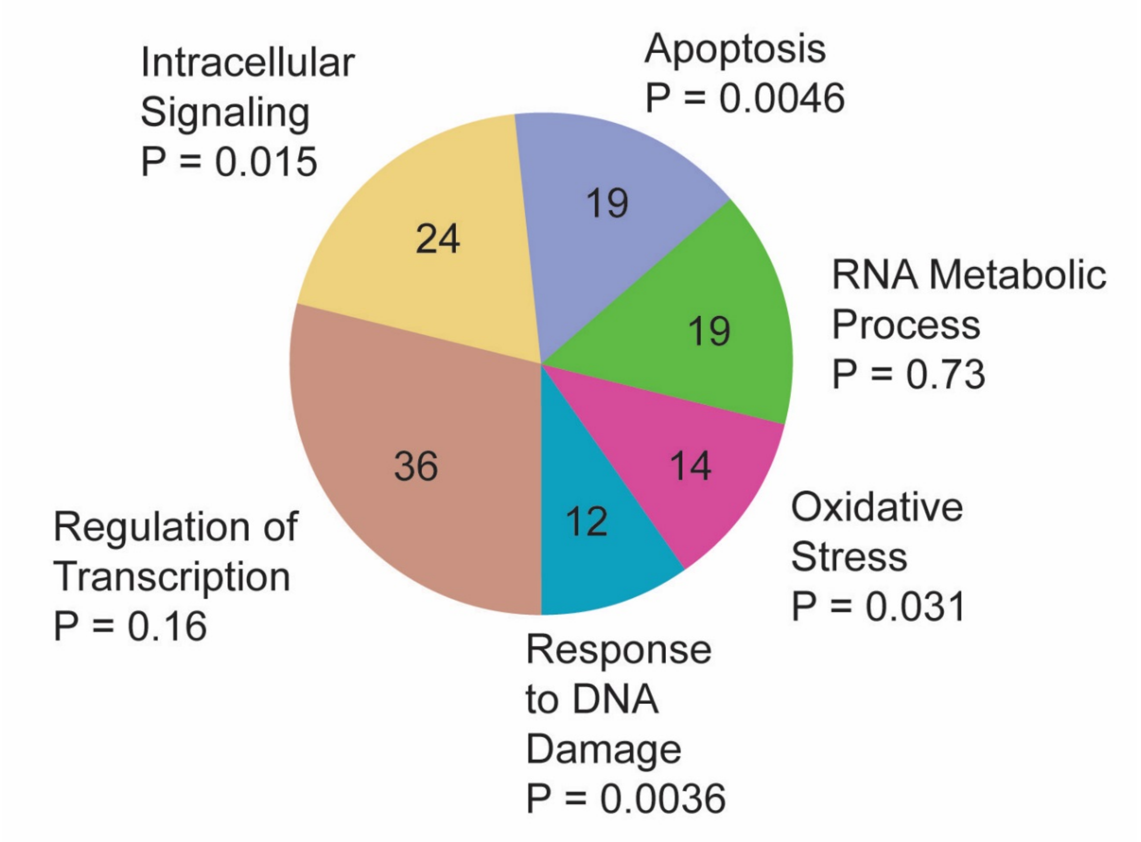

Supplementary Figure 1. Gene Ontology (GO) analysis of the 229 survival associated alternative splicing events, using GO biological process (BP) functional categories. The six largest BP functional categories are shown in the figure with their respective enrichment *P*-values.

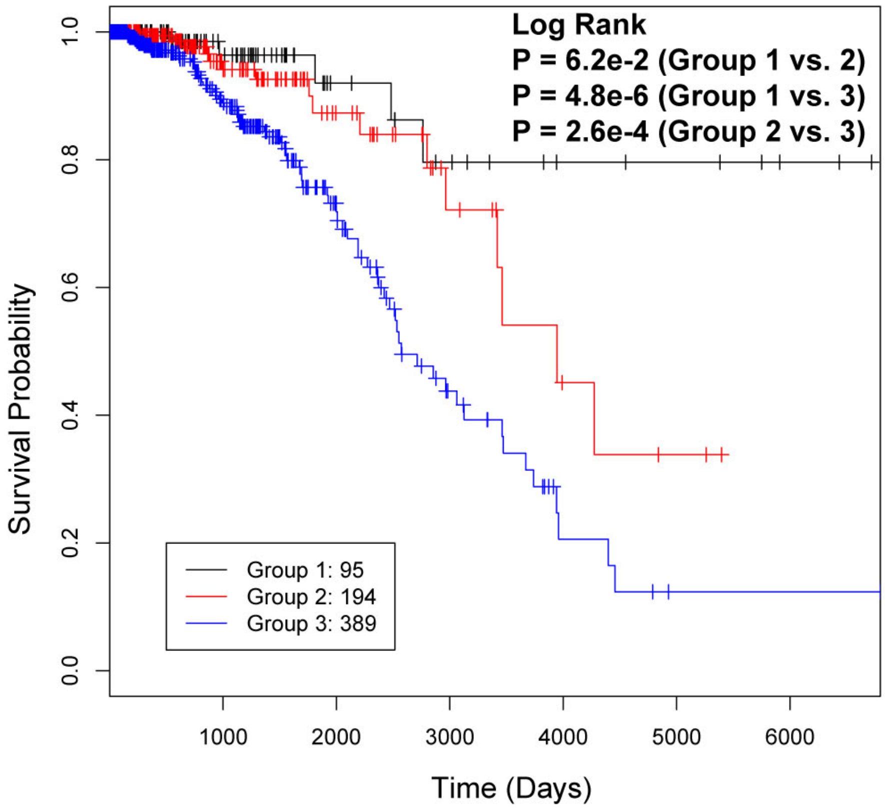

Supplementary Figure 2. Kaplan-Meier survival plots of the three IDC patient subgroups identified by hierarchical clustering of 176 survival associated alternative exons (P ≤ 0.01; SURVIV analysis of all IDC patients).

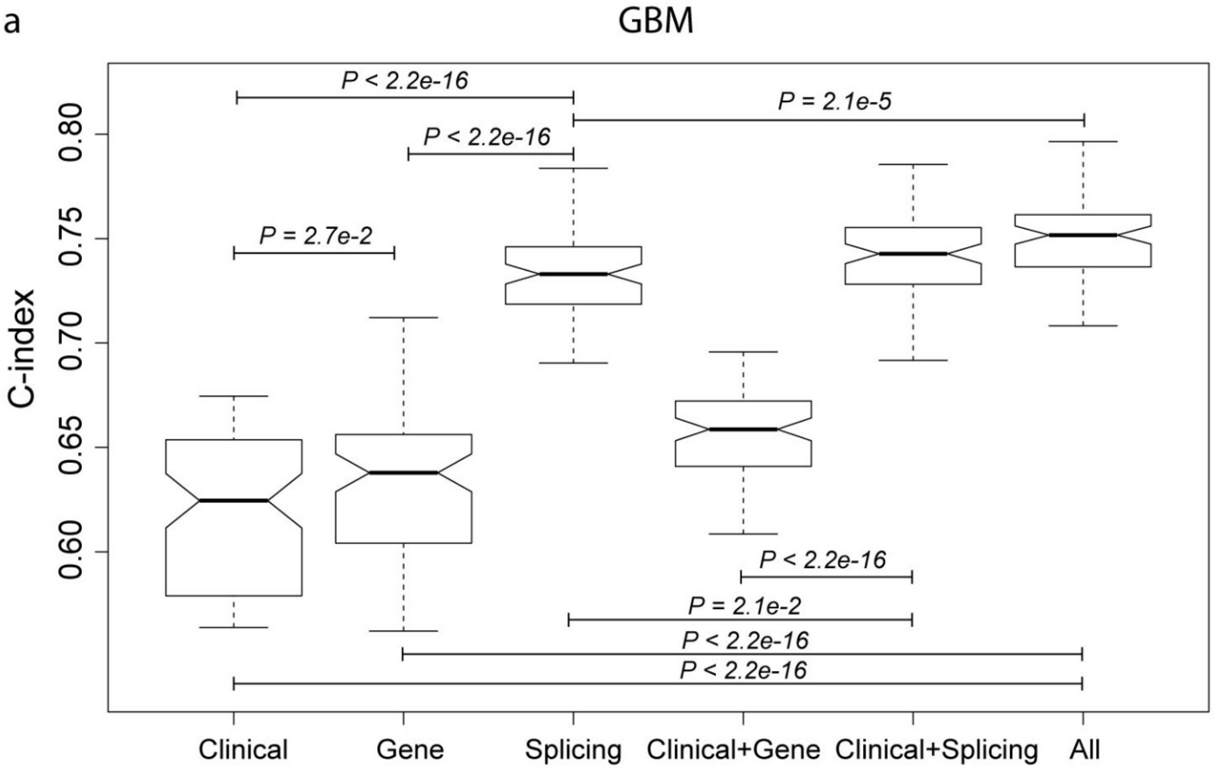

Supplementary Figure 3a. Cross-validation of different classes of survival predictors in GBM measured by the C-index. The center value of the box plot is the median C-index from 100 rounds of cross-validation. The notch represents the 95% confidence interval of the median.

b

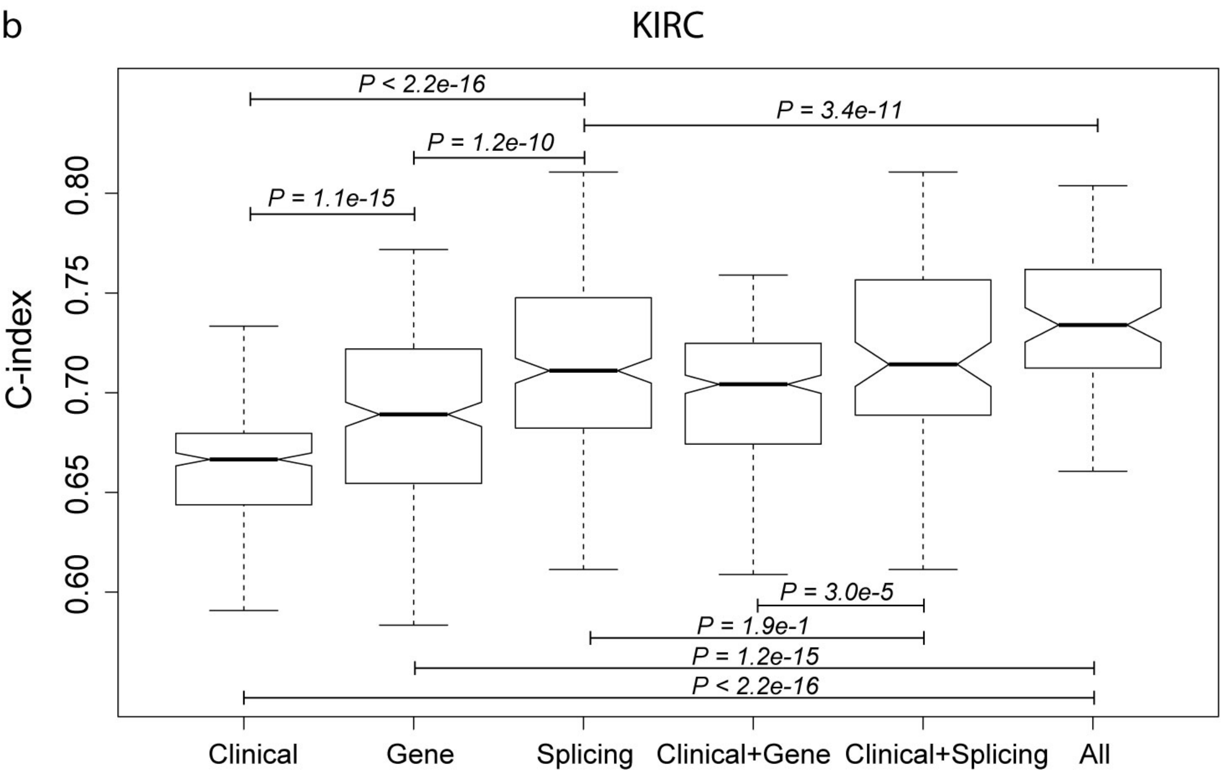

Supplementary Figure 3b. Cross-validation of different classes of survival predictors in KIRC measured by the C-index. The center value of the box plot is the median C-index from 100 rounds of cross-validation. The notch represents the 95% confidence interval of the median.

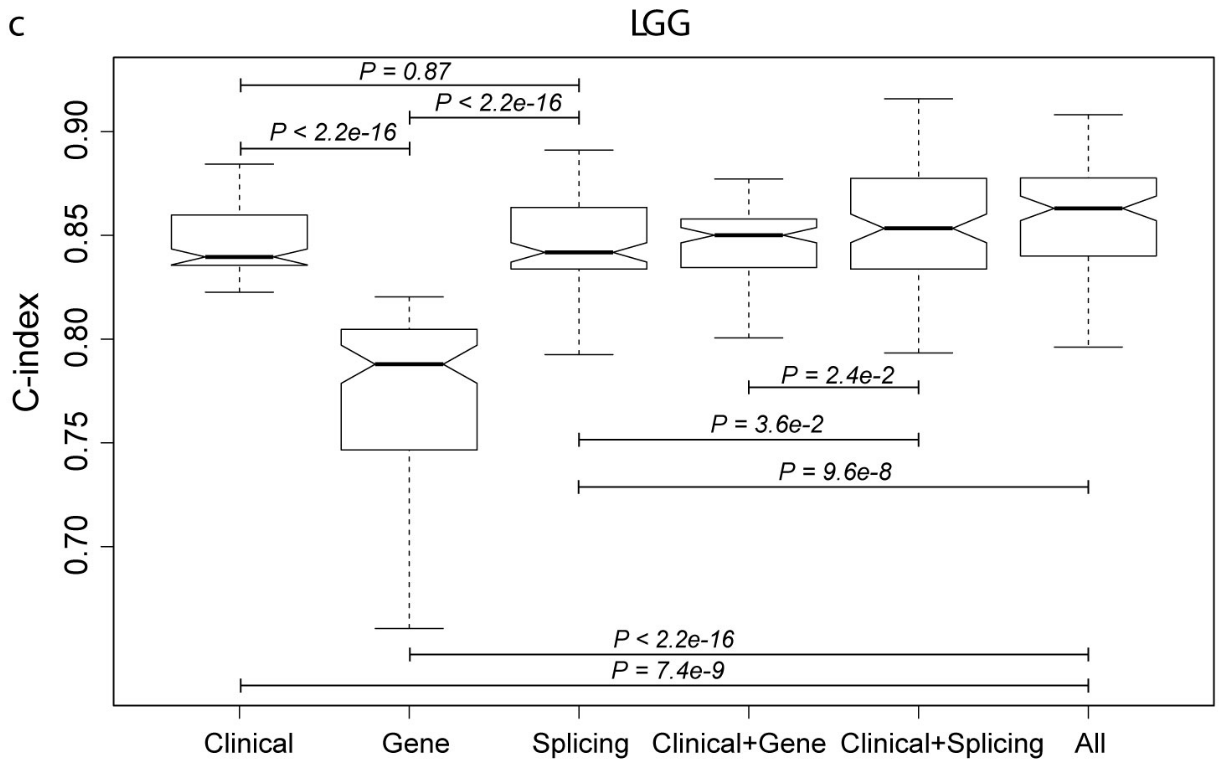

Supplementary Figure 3c. Cross-validation of different classes of survival predictors in LGG measured by the C-index. The center value of the box plot is the median C-index from 100 rounds of cross-validation. The notch represents the 95% confidence interval of the median.

d

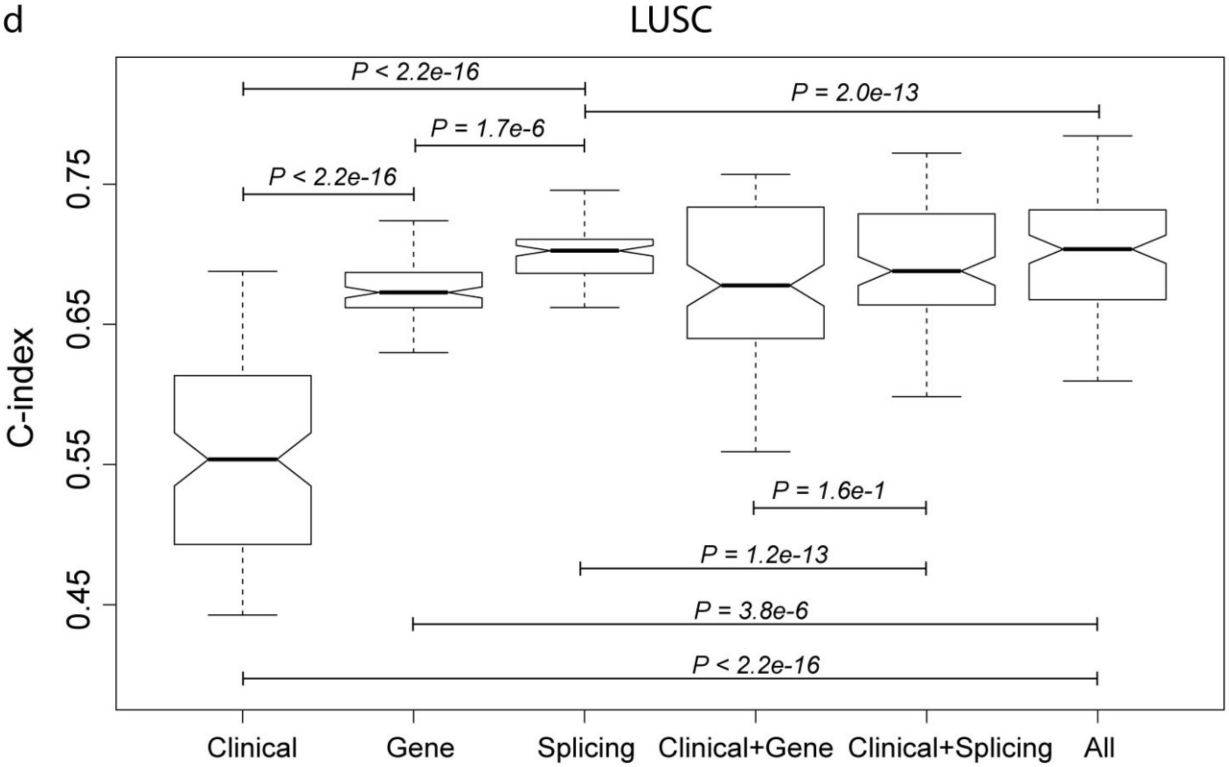

Supplementary Figure 3d. Cross-validation of different classes of survival predictors in LUSC measured by the C-index. The center value of the box plot is the median C-index from 100 rounds of cross-validation. The notch represents the 95% confidence interval of the median.

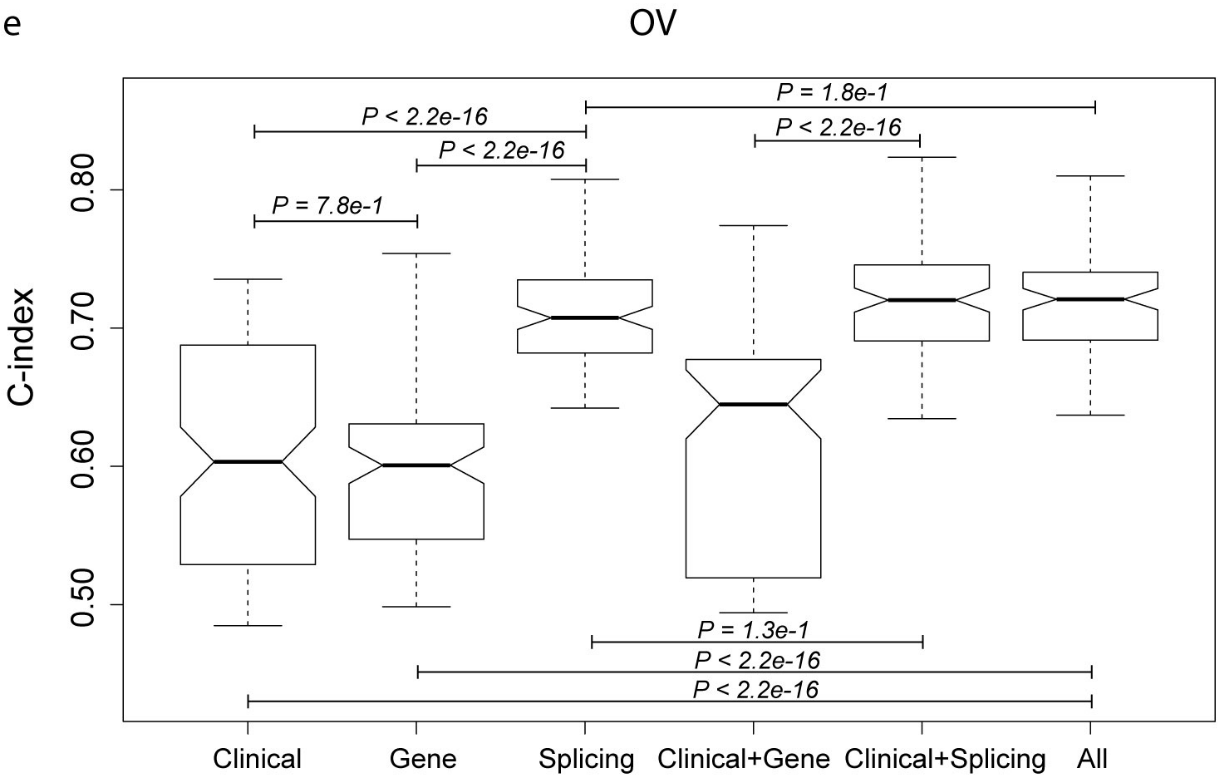

Supplementary Figure 3e. Cross-validation of different classes of survival predictors in OV measured by the C-index. The center value of the box plot is the median C-index from 100 rounds of cross-validation. The notch represents the 95% confidence interval of the median.

Supplementary Methods

1. RNA-seq measurement of mRNA isoform variation.

In this document, we will use the exon skipping type of alternative splicing events to illustrate the SURVIV statistical model. However, we note that the same statistical framework can be applied to other types of alternative splicing and mRNA isoform variation patterns. Briefly, RNA-seq reads are mapped to both the genome and splice junctions. For an exon skipping event, the exon inclusion level (denoted as Percent Spliced In, or ψ) is calculated using the numbers of reads uniquely mapped to the exon inclusion isoform or the exon skipping isoform. The number of reads mapped to the exon inclusion isoform is denoted as IC. The number of reads mapped to the exon skipping isoform is denoted as SC. The total number of reads mapped to the exon inclusion or skipping isoform is denoted as n, as $n = IC + SC$. In Fig. 1C, the reads of the exon inclusion isoform and the exon skipping isoform are illustrated for an exon skipping event. Similarly, we can count the isoform-specific reads for other types of alternative splicing and mRNA isoform variation patterns.

As the lengths of isoform-specific segments may differ between alternative mRNA isoforms (e.g. between exon inclusion versus skipping isoforms), we need to normalize the isoform-specific read counts by the effective lengths of isoform-specific segments in calculating the exon inclusion levels. For a segment whose length is l, and the length of the read is r, the effective length of the segment is defined by the number of unique read intervals in this region, which is $l - r + 1$. For an exon skipping event, we denote the effective length of the exon inclusion isoform as l_I, and the effective length of the exon skipping isoform as l_S. Adjusted by the effective lengths of the isoform-specific segments, the exon inclusion level ψ can be estimated as $\hat{\psi} = \frac{IC / l_I}{IC / l_I + SC / l_S}$. Assuming the read count from the exon inclusion isoform follows a binomial distribution, the total read count $n = IC + SC$, and the proportion of reads from the exon inclusion isoform is $p = \frac{l_I \psi}{l_I \psi + l_S (1 - \psi)}$, then:

$$IC \mid \psi \sim Binomial(n = IC + SC, p = \frac{l_I \psi}{l_I \psi + l_S (1 - \psi)}) \quad (1)$$

This binomial model defines the relationship among the exon inclusion reads, exon skipping reads, and the exon inclusion levels in individual samples, adjusted by the effective lengths of the exon inclusion and skipping isoforms.

This statistical model can be readily applied to isoform-specific count data generated by other RNA-seq alignment and read counting pipelines, although we do not expect any notable difference in the accuracy of PSI quantification as illustrated recently through comprehensive evaluations of multiple pipelines[1]. In future work, it is possible to extend the binomial distribution in SURVIV to a multinomial distribution, which will allow us to study complex events involving more than two isoform choices from a local alternatively spliced region, or to analyze the abundance distribution of all (N>2) full-length isoforms of a gene.

2. SURVIV statistical model.

In this section, we introduce the SURVIV statistical model that tests the association between mRNA isoform ratio and patient survival time. For an exon skipping event, SURVIV models the association between exon inclusion level and patient survival time. However, the challenge is that in RNA-seq data, exon inclusion levels are not directly observable but instead are estimated from RNA-seq read counts. The estimation uncertainty of exon inclusion level therefore depends on the RNA-seq read coverage for the event of interest in a given RNA sample, with a higher read coverage leading to a more reliable estimate. A key feature of SURVIV is that it models the estimation uncertainty of mRNA isoform ratio in the survival analysis, using the binomial model described in the previous section.

Specifically, we use the proportional hazards model to assess the association between exon inclusion level and patient survival time. The proportional hazards model assumes that the hazard rate λ_k of patient k is a function of the exon inclusion level ψ_k :

$$\lambda_k(t) = \lambda_0(t)\exp(\beta\,\mathrm{logit}(\psi_k)). \tag{2}$$

In Equation (2), $\mathrm{logit}(\psi_k)$ is the logit transformation of the exon inclusion level ψ_k, which is a fraction number between 0 to 1. $\lambda_0(t)$ is the baseline hazard function that defines the hazard rate

at time t when $\text{logit}(\psi_k) = 0$. β is the survival coefficient that represents the effect of exon inclusion level on hazard rate. The baseline hazard function is identical for all the patients.

Each patient's exon inclusion level ψ_k is associated with the patient's observed survival data. For patient k, let D_k denote the time to event (i.e. death), C_k be the censoring time and δ_k be the event (death) indicator. $\delta_k = 1$ if $D_k \le C_k$, otherwise $\delta_k = 0$. For the right-censored data in the survival analysis, the observed survival data include the event indicator δ_k and follow-up time $t_k = \min(D_k, C_k)$. The likelihood function of the proportional hazards model is used to model censoring data[2]:

$$P(\delta_k, t_k; \beta, \lambda_0 \mid \psi_k) = \exp(\delta_k \log \lambda_0(t_k) + \delta_k \beta \,\text{logit}(\psi_k) - \Lambda_0(t_k) \exp(\beta \,\text{logit}(\psi_k))). \tag{3}$$

In which, β is the survival coefficient that represents the effect of exon inclusion level on hazard rate. λ_0 is the baseline hazard function. Λ_0 is the cumulative baseline hazard function.

In RNA-seq data, exon inclusion levels ψ_k are not directly observable but instead are estimated from RNA-seq read counts of the exon inclusion isoform IC_k and the exon skipping isoform SC_k. Therefore we have:

$$\begin{aligned} &IC_k \mid \psi_k \sim Binomial(n_k = IC_k + SC_k, p_k = \frac{l_I \psi_k}{l_I \psi_k + l_S(1-\psi_k)}). \\ &P(IC_k \mid \psi_k) = \exp(\log \binom{n_k}{IC_k} + IC_k \log(\frac{l_I \psi_k}{l_I \psi_k + l_S(1-\psi_k)}) + (n_k - IC_k) \log(1 - \frac{l_I \psi_k}{l_I \psi_k + l_S(1-\psi_k)})). \end{aligned} \tag{4}$$

In order to model the estimation uncertainty of exon inclusion level ψ_k in the survival analysis, we use the survival measurement error framework[3] to combine the survival model (3) and the model for RNA-seq estimation uncertainty (4), along with the non-informative prior that if not estimated by RNA-seq, the exon inclusion level can be any value from 0 to 1, i.e. $P(\psi_k) = uniform(0,1)$.

The joint full likelihood function of our model is proportional to

$$\prod_k P(\delta_k, t_k, IC_k, \psi_k; \beta, \lambda_0) = \prod_k P(\delta_k, t_k; \beta, \lambda_0 \mid \psi_k) P(IC_k \mid \psi_k) P(\psi_k)$$

$$\propto \prod_k \exp(\delta_k \log \lambda_0(t_k) + \delta_k \beta \operatorname{logit}(\psi_k) - \Lambda_0(t_k) \exp(\beta \operatorname{logit}(\psi_k)) + IC_k \log(\frac{l_I \psi_k}{l_I \psi_k + l_S(1-\psi_k)}) \qquad (5)$$

$$+ (n_k - IC_k) \log(1 - \frac{l_I \psi_k}{l_I \psi_k + l_S(1-\psi_k)})).$$

The difference between the model we use in Equation (5) and a standard proportional hazards model is that our model considers the estimation uncertainty of exon inclusion level in the RNA-seq data.

We should emphasize that the SURVIV model is conceptually distinct from previous methods and models on measurement errors in survival analysis, which were mainly concerned with continuous Gaussian errors. By contrast, our model deals with indirect observations (i.e. isoform-specific counts) in RNA-seq inference of alternative isoform variation, and the relationship between these observations and the variables to be inferred (i.e. isoform proportions) is more complex than simple additive Gaussian errors.

3. Parameter estimation of the SURVIV statistical model.

The full likelihood of the SURVIV model (Equation (5)) contains the latent variable ψ_k, which is not directly observable from data. In order to estimate the survival coefficient β and the baseline hazard function λ_0, we will remove the latent variable ψ_k by integral:

$$P(\delta_k, t_k, IC_k; \beta, \lambda_0) = \int_0^1 P(\delta_k, t_k, IC_k, \psi_k; \beta, \lambda_0) d\psi_k.$$

Since there is no closed-form solution for the integral, Laplace approximation is used here for the numerical integration of the latent variable ψ_k.

The Laplace approximation of the integral of ψ_k is:

$$
\begin{aligned}
&\int_0^1 P(\delta_k, t_k, IC_k, \psi_k; \beta, \lambda_0) d\psi_k \\
&\overset{f=\log P}{=} \int_0^1 \exp(f(\delta_k, t_k, IC_k, \psi_k; \beta, \lambda_0)) d\psi_k \\
&\overset{x_k=\mathrm{logit}(\psi_k)}{=} \int_{-\infty}^{+\infty} \exp(\tilde{f}(\delta_k, t_k, IC_k, x_k; \beta, \lambda_0)) dx_k \qquad (6) \\
&= \int_{-\infty}^{+\infty} \exp(\tilde{f}(\delta_k, t_k, IC_k, \hat{x}_k; \beta, \lambda_0) + 0.5 \frac{\partial^2 \tilde{f}(\delta_k, t_k, IC_k, \hat{x}_k; \beta, \lambda_0)}{\partial x_k^2} (x_k - \hat{x}_k)^2 + o((x_k - \hat{x}_k)^2)) dx_k \\
&\approx 2\pi(|\frac{\partial^2 \tilde{f}(\delta_k, t_k, IC_k, \hat{x}_k; \beta, \lambda_0)}{\partial x_k^2}|)^{-0.5} \exp(\tilde{f}(\delta_k, t_k, IC_k, \hat{x}_k; \beta, \lambda_0)).
\end{aligned}
$$

In Equation (6), the Laplace's method approximates the distribution of $x_k = \mathrm{logit}(\psi_k)$ by a normal distribution, using the second-level derivative function in the Taylor series. $\tilde{f}$ is the log likelihood function after logit parameter transformation of latent variable ψ_k. The first-level derivative function of the Taylor series is equal to zero because $\hat{x}_k$ is the maximum likelihood estimate based on the full likelihood function:

$$
\begin{aligned}
&\hat{x}_k = \underset{x_k}{\mathrm{argmax}}(\tilde{f}(\delta_k, t_k, IC_k, x_k; \beta, \lambda_0)), \\
&\hat{\psi}_k = \mathrm{logit}^{-1}(\hat{x}_k).
\end{aligned}
$$

In Equation (6), the secondary derivative function of the log likelihood function $\tilde{f}$ is:

$$
\frac{\partial^2 \tilde{f}(\delta_k, t_k, IC_k, \hat{x}_k; \beta, \lambda_0)}{\partial x_k^2} = F(\hat{\psi}_k)(1 - \beta^2 \Lambda_0(t_k) \exp(\beta\, \mathrm{logit}(\hat{\psi}_k)) / F(\hat{\psi}_k)),
$$

$$
\begin{aligned}
F(\hat{\psi}_k) = {} & 2\hat{\psi}_k^2 - 2\hat{\psi}_k - \hat{\psi}_k^2(1-\hat{\psi}_k)^2 \{IC_k l_S \frac{(2l_I + l_S)\hat{\psi}_k + l_S(1-\hat{\psi}_k)}{\hat{\psi}_k^2 (l_I \hat{\psi}_k + l_S(1-\hat{\psi}_k))^2} + SC_k l_I \frac{(l_I + 2l_S)(1-\hat{\psi}_k) + l_I \hat{\psi}_k}{(1-\hat{\psi}_k)^2 (l_I \hat{\psi}_k + l_S(1-\hat{\psi}_k))^2}\} \\
& + \hat{\psi}_k(1-\hat{\psi}_k)(1-2\hat{\psi}_k)\{\frac{IC_k l_S}{\hat{\psi}_k (l_I \hat{\psi}_k + l_S(1-\hat{\psi}_k))} - \frac{SC_k l_I}{(1-\hat{\psi}_k)(l_I \hat{\psi}_k + l_S(1-\hat{\psi}_k))}\}.
\end{aligned}
$$

After the Laplace approximation in Equation (6), the log likelihood function becomes:

$$
\begin{aligned}
&\sum_k \log P(\delta_k, t_k, IC_k; \beta, \lambda_0) \\
&= C + \sum_k \{-0.5 * \log(1 - \beta^2 \Lambda_0(t_k) \exp(\beta\, \mathrm{logit}(\hat{\psi}_k)) / F(\hat{\psi}_k)) \qquad (7) \\
&+ \delta_k \log \lambda_0(t_k) + \delta_k \beta\, \mathrm{logit}(\hat{\psi}_k) - \Lambda_0(t_k) \exp(\beta\, \mathrm{logit}(\hat{\psi}_k))\}.
\end{aligned}
$$

In which, C includes the constant that does not affect the parameter estimation of the survival coefficient β and the baseline hazard function λ_0.

4. Optimization procedure and likelihood-ratio test for the model.

In this section, we describe the optimization procedure to calculate the maximum likelihood estimation (MLE) of the survival coefficient β and the baseline hazard function λ_0 based on Equation (7). Equation (7) has a closed form solution given the fixed latent variable $\hat{\psi}_k$. However, the estimation of the latent variable depends on fixed values of $\hat{\beta}$ and $\hat{\lambda}_0$. Therefore, we use an iterative procedure for the MLE calculation.

The initial value of the latent variable $\hat{\psi}_k$ is derived from the individual binomial distribution of each patient: $\hat{\psi}_k^{(1)} = \dfrac{IC_k / l_I}{IC_k / l_I + (n_k - IC_k) / l_S}$. The initial value of the baseline hazard function $\hat{\lambda}_0^{(1)}$ and the baseline cumulative hazard function $\hat{\Lambda}_0^{(1)}$ are set to be 1.

In each round r of the iterative optimization procedure, we first estimate the MLE of the survival coefficient β, based on the estimated values of other parameters from the last round (*r*-1): latent variable $\hat{\psi}_k^{(r-1)}$, baseline hazard function $\hat{\lambda}_0^{(r-1)}$, baseline cumulative hazard function $\hat{\Lambda}_0^{(r-1)}$, by searching for the β that optimizes the marginal distribution in Equation (7).

$$
\begin{aligned}
\hat{\beta}^{(r)} &= \underset{\beta}{\operatorname{argmax}} \sum_k \log P(\delta_k, t_k, IC_k; \beta, \lambda_0) \\
&= \underset{\beta}{\operatorname{argmax}} \sum_k \{-0.5 * \log(1 - \beta^2 \hat{\Lambda}_0(t_k) \exp(\beta \operatorname{logit}(\hat{\psi}_k^{(r-1)})) / F(\hat{\psi}_k^{(r-1)})) \\
&+ \delta_k \log \hat{\lambda}_0^{(r-1)}(t_k) + \delta_k \beta \operatorname{logit}(\hat{\psi}_k^{(r-1)}) - \hat{\Lambda}_0(t_k) \exp(\beta \operatorname{logit}(\hat{\psi}_k^{(r-1)}))\}
\end{aligned}
$$

After we estimate the survival coefficient $\hat{\beta}^{(r)}$, we then estimate the baseline hazard function $\lambda_0^{(r)}$ and baseline cumulative hazard function $\Lambda_0^{(r)}$. Note that with increasing number of read count n_k, $1/F(\hat{\psi}_k)$ will converge to 0. Therefore, we use Taylor expansion to approximate the log component of Equation (7):

$$
\begin{aligned}
&\sum_k \log P(\delta_k, t_k, IC_k; \hat{\beta}^{(r)}, \lambda_0) \\
&= C + \sum_k \{-0.5 * \log(1-(\hat{\beta}^{(r)})^2 \Lambda_0(t_k) \exp(\hat{\beta}^{(r)} \operatorname{logit}(\hat{\psi}_k^{(r-1)})) / F(\psi_k^{(r-1)})) \\
&+ \delta_k \log \lambda_0(t_k) + \delta_k \hat{\beta}^{(r)} \operatorname{logit}(\hat{\psi}_k^{(r-1)}) - \Lambda_0(t_k) \exp(\hat{\beta}^{(r)} \operatorname{logit}(\hat{\psi}_k^{(r-1)}))\} \\
&\approx C + \sum_k \{\delta_k \log \lambda_0(t_k) + \delta_k \hat{\beta}^{(r)} \operatorname{logit}(\hat{\psi}_k^{(r-1)}) \\
&- \Lambda_0(t_k)(1-0.5(\hat{\beta}^{(r)})^2 / F(\hat{\psi}_{ik}^{(r-1)})) \exp(\hat{\beta}^{(r)} \operatorname{logit}(\hat{\psi}_k^{(r-1)}))\}.
\end{aligned}
$$

Compared to the conventional proportional hazards model, an extra weight $(1-0.5(\hat{\beta}^{(r)})^2 / F(\hat{\psi}_k^{(r-1)}))$ is added to the last term of the survival likelihood. Thus, the baseline hazard function $\lambda_0^{(r)}$ can now be estimated by a weighted Nelson-Aalen estimator[4].

$$
\lambda_0^{(r)} = \frac{d_k}{\sum_{k \in R(t_k)} (1-0.5(\hat{\beta}^{(r)})^2 / F(\hat{\psi}_k^{(r-1)})) \exp(\hat{\beta}^{(r)} \operatorname{logit}(\hat{\psi}_k^{(r-1)})}.
$$

In which, d_k is the number of events (death) at time t_k, and $R(t_k)$ is the risk set at time t_k, which includes all patients who are event-free (alive) before time t_k. After that, we update the estimated value of the latent variable $\hat{\psi}_k^{(r)}$.

$$
\hat{\psi}_k^{(r)} = \underset{\psi_k}{\operatorname{argmax}}(\log P(\delta_k, t_k, IC_k, \psi_k; \beta^{(r)}, \lambda_0^{(r)})).
$$

This optimization procedure iterates for multiple rounds until the difference in log likelihood between two consecutive iterations is smaller than 10^{-4}.

We use a likelihood-ratio test to calculate the P-value of significant association between exon inclusion level and patient survival time. The null hypothesis is that there is no association between exon inclusion level and patient survival time, i.e. $H_0 : \beta = 0$. The likelihood-ratio test compares the log-likelihood under the constraint of the null hypothesis ($\log L_{\beta=0}$) and the likelihood without constraint ($\log L$):

$$
-2(\log L_{\beta=0} - \log L) \sim \chi_1^2
$$

With such a null hypothesis, the likelihood-ratio test statistic asymptotically follows a chi-squared distribution with one degree of freedom. The constrained MLE is estimated with the same procedure as described above, except for the additional constraint of $\beta = 0$.

Supplementary References

1. Alamancos GP, Pages A, Trincado JL, Bellora N, Eyras E. Leveraging transcript quantification for fast computation of alternative splicing profiles. *RNA* **21**, 1521-1531 (2015).

2. Hu P, Tsiatis AA, Davidian M. Estimating the parameters in the Cox model when covariate variables are measured with error. *Biometrics* **54**, 1407-1419 (1998).

3. Prentice RL. Covariate Measurement Errors and Parameter-Estimation in a Failure Time Regression-Model. *Biometrika* **69**, 331-342 (1982).

4. Breslow N. Covariance analysis of censored survival data. *Biometrics* **30**, 89-99 (1974).
